# Efficacy and safety of atogepant, a small molecule CGRP receptor antagonist, for the preventive treatment of migraine: a systematic review and meta-analysis

**DOI:** 10.1186/s10194-024-01822-2

**Published:** 2024-07-19

**Authors:** Min Hou, Xiaofeng Luo, Shuangshuang He, Xue Yang, Qing Zhang, Meihua Jin, Pan Zhang, Yang Li, Xiaoting Bi, Juan Li, Caiyi Cheng, Qiang Xue, Haiyan Xing, Yao Liu

**Affiliations:** 1 Department of Pharmacy, Daping Hospital, Army Medical University, Chongqing, 400042 P. R. China; 2 Department of Pharmacy, Chongqing Medical and Pharmaceutical College, Chongqing, 401331 P. R. China

**Keywords:** Migraine, Atogepant, CGRP receptor antagonists, Meta-analysis

## Abstract

**Background:**

Migraine is one of the most common diseases worldwide while current treatment options are not ideal. New therapeutic classes of migraine, the calcitonin gene-related peptide (CGRP) antagonists, have been developed and shown considerable effectiveness and safety. The present study aimed to systematically evaluate the efficacy and safety of atogepant, a CGRP antagonist, for migraine prophylaxis from the results of randomized controlled trials (RCTs).

**Methods:**

The Cochrane Library, Embase, PubMed and https://www.clinicaltrials.gov/ were searched for RCTs that compared atogepant with placebo for migraine prophylaxis from inception of the databases to Feb 1, 2024. Outcome data involving efficacy and safety were combined and analyzed using Review Manager Software version 5.3 (RevMan 5.3). For each outcome, risk ratios (RRs) or standardized mean difference (SMD) were calculated.

**Results:**

4 RCTs with a total of 2813 subjects met our inclusion criteria. The overall effect estimate showed that atogepant was significantly superior to placebo in terms of the reduction of monthly migraine (SMD − 0.40, 95% CI -0.46 to -0.34) or headache (SMD − 0.39, 95% CI -0.46 to -0.33) days, the reduction of acute medication use days (SMD − 0.45, 95% CI -0.51 to -0.39) and 50% responder rate (RR 1.66, 95% CI 1.46 to 1.89), while no dose-related improvements were found between different dosage groups. For the safety, significant number of patients experienced treatment-emergent adverse events (TEAEs) with atogepant than with placebo (RR 1.10, 95% CI 1.02–1.21) while there was no obvious difference between the five dosage groups. Most TEAEs involved constipation (RR 2.55, 95% CI 1.91–3.41), nausea (RR 2.19, 95% CI 1.67–2.87) and urinary tract infection (RR 1.49, 95% CI 1.05–2.11). In addition, a high dosage of atogepant may also increase the risk of treatment-related TEAEs (RR 1.64, 95% CI 1.02–2.63) and fatigue (RR 3.07, 95% CI 1.13–8.35).

**Conclusions:**

This meta-analysis suggests that atogepant is effective and tolerable for migraine prophylaxis including episodic or chronic migraine compared with placebo. It is critical to weigh the benefits of different doses against the risk of adverse events in clinical application of atogepant. Longer and multi-dose trials with larger sample sizes are required to verify the current findings.

**Supplementary Information:**

The online version contains supplementary material available at 10.1186/s10194-024-01822-2.

## Background

Migraine is a complex, variable neurological disease that characterized by recurrent disabling attacks of headache associated with photophobia, phonophobia, nausea, and vomiting [1]. It was ranked second overall in global disability and even topped the list in young women in the global burden disease of 2019 [2, 3].

Migraine prophylaxis is primarily aimed at reducing attack frequency, severity, duration, and disability, avoiding the risk of medication overuse and improving health-related quality of life [4]. However, according to statistics, the usage rates for preventive treatments in patients with episodic or chronic migraine is lower, and usually less than half the patients [5, 6]. Normally one main reason is that the low efficacy and poor tolerability result in treatment failure and discontinuation, and poor satisfaction overall exists among those people for whom preventive treatments have failed [5, 7]. Also, the healthcare resource use and costs is another important factor [8]. In recent years, monoclonal antibodies targeting the CGRP pathway and the new generations of oral CGRP receptor antagonists were developed and applied in the prophylaxis and treatment of migraine [9]. These agents tried to address the limitations of traditional migraine medications for treatment. Nevertheless, the first generation of CGRP receptor antagonists was discontinued due to hepatotoxicity [10].

Atogepant, a new specific medication targeted CGRP receptor, had demonstrated efficacy and tolerability for migraine prophylaxis in the phase I/II randomized clinical trials (RCTs). Different doses of atogepant had been approved by the FDA and EMA for migraine prophylaxis. In theory, the smallest effective dose is more economical for patients and may have better safety. What about the effectiveness and safety of different doses of atogepant? It is necessary to conduct a systematic review and meta-analysis comparing the efficacy and safety of different dosages of atogepant for migraine prophylaxis.

Up to now, there were two systematic review that examined the differences between atogepant and placebo in the efficacy and tolerability for the preventive treatment of migraine [11, 12]. However, some of their results were different in the two previous studies. Furthermore, these studies were based on the small sample sizes, and the conclusions drawn may not be helpful for clinicians to make treatment decisions and provide guidance. In nearly a year, two large RCTs [13, 14] were released after the publication of the above two systematic review [11, 12], and it is essential to update the existing results and provide a more robust and comprehensive review based on the latest evidence. Therefore, the present study was to perform a systematic review and meta-analysis to further evaluate and verify the safety and efficacy of atogepant in the treatment of migraine prevention.

## Methods

### Literature search and inclusion criteria

Two of us (MH and XFL) independently searched Cochrane Library, Embase, PubMed and https://www.clinicaltrials.gov/ for RCTs from inception of the databases to Feb 1, 2024. The following Medical Subject Headings (MeSH) and keywords were used for the literature retrieval: “migraine” and “CGRP receptor antagonist” or “atogepant (AGN-241689)”and “randomized clinical trials (RCTs)”. Then, we thoroughly examined all potentially relevant articles, including their reference lists, to ensure that no relevant studies were overlooked. Studies that satisfied these criteria were accepted: (1) RCTs assessing the efficacy and safety of atogepant for the migraine prophylaxis; (2) migraine sufferers (18 years of age or older) with or without aura were included in the study; (3) atogepant in any dosage or formulation as treatment group, and placebo as control group, respectively; (4) the efficacy and safety outcomes were provided or could be calculated from original data in these articles. In addition, studies involving the combination of atogepant with other drugs in the intervention group will be excluded. When there were disagreements between the two researchers, they were settled by agreement or, if required, by discussing with the third author (YL or HYX or QX).

### Evaluation of risk of bias and quality of evidence

Two of us (MH and SSH) independently evaluated the methodological quality of these studies included using Review Manager Software version 5.3 (RevMan 5.3) provided by the Cochrane Collaboration [15]. Detailed criteria involved seven-item scale for making judgments about the risk of bias from each of the items in the tool are available [16], and risk ratings of high, low, or unclear were judged for the included trials. Discrepancies between the two authors were discussed and resolved by consensus or consultation with the third reviewer (XY or QZ). Besides, the two reviewers also independently assessed the strength of the current evidence for each outcome using the Grading of Recommendations, Assessment, Development, and Evaluation working group (GRADE) tool across five domains, including overall risk of bias, inconsistency, indirectness, imprecision, and publication bias [17, 18]. Finally, the overall quality of evidence was summarized across domains using the 4 confidence levels of the GRADE approach: very low, low, moderate, or high [18].

### Statistical analysis

The efficacy and safety of CGRP receptor antagonists for migraine prophylaxis were pooled by standardized mean difference (SMD) or risk ratios (RRs) with 95% confidence intervals (CI) through either a fixed- or random-effect model, which was performed using RevMan 5.3 (Cochrane Collaboration, Oxford, England). The original data from the graphs were extracted with the GetData Graph Digitizer software (version 2.26).For the heterogeneity analysis, the *I*^*2*^ index was used to estimate it and automatically calculated by the RevMan 5.3 software [19–21]. *I*^*2*^ values are acceptable if they are less than 50%, which denotes non-significant heterogeneity. In that case, the fixed-effect model of analysis is appropriate. If not, the random-effect model is taken into account [22].

## Results

### Selection and inclusion of studies

**Fig. 1:**
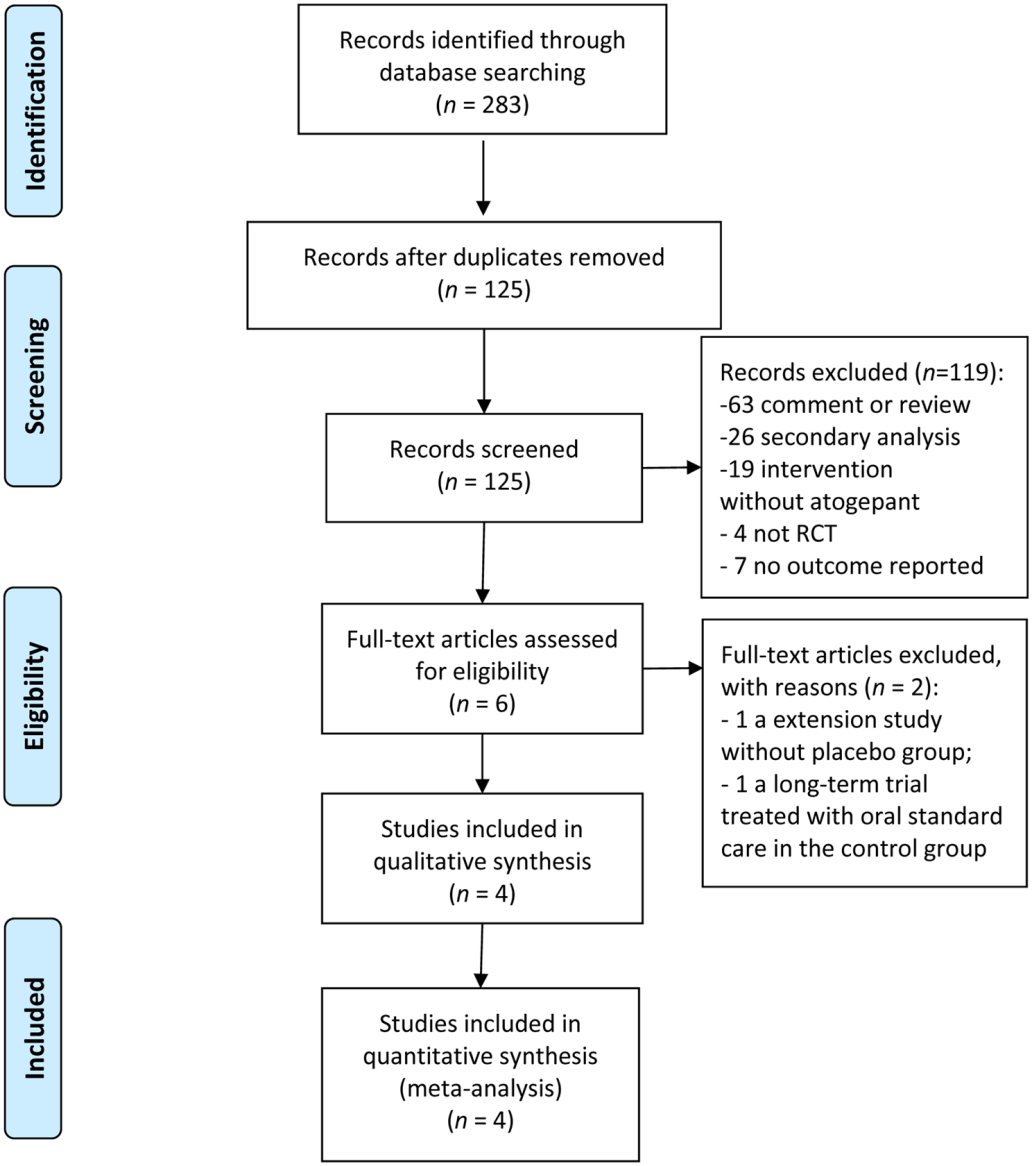
Process of identifying eligible studies for the meta-analysis

A total of 283 articles were yielded based on the initial search strategy, then 158 duplicates were removed during title screening. 125 potentially relevant studies remained. Of these, 6 articles were reviewed in the full text, and 4 RCTs involved in phase II – III (2813 participants) were considered eligible and were taken into this analysis [13, 14, 23, 24], which included an additional 2 RCTs with 1064 patients on the basis of previous meta-analysis [11, 12]. A flow chart outlining the literature search is shown in Fig. 1.

All studies involved a placebo comparator, the agents studied were different doses of atogepant. The baseline demographics, specifically age and gender, did not differ widely among the included studies. Participants were adults (≥ 18 years) with a mean age of 41.34 years in the atogepant group and 41.47 years in the placebo group, and reported gender was 2472 females and 341 males with female-to-male ratio of 7.25:1. Almost all migraine sufferers with or without aura had been diagnosed according to the International Classification of Headache Disorders (ICHD) 3/3-beta [25, 26]. Patients in the atogepant group had suffered from migraine with a mean frequency of 10.7 days per month, while those in the placebo group reported a mean frequency of 11.5 migraine days per month. Details of the study characteristics were shown in Table 1.

**Table 1:** Characteristics of the included studies

Included trials	Location(s); Study design	Eligibility criteria	Gender(male/female) mean age (years)		Migraine attacks per month		Medication dosage	Study period	Efficacy outcomes		Safety outcomes
			Control	Trial	Control	Trial			Primary	Secondary	
Goadsby P J et al., 2020	Multinational RCT	ICHD-3 beta	32/154; 40.5± 11.7	79/560; 40.07± 12.33	7·8 ± 2·5	7.6 ± 2.5	10 mg QD 30 mg QD 60 mg QD 30 mg BID 60 mg BID	12Weeks	MMDs	MHDs; acute medication use days; ≥50% reduction in MMDs	TEAEs; treatment-related TEAEs; serious TEAEs; treatment-related serious TEAE; nausea; upper respiratory tract infection; nasopharyngitis; constipation; urinary tract infection; fatigue
Ailani J et al., 2021	Multinational RCT	ICHD-3	24/198; 40.3± 12.8	77/603; 42.01± 12.03	7.5 ± 2.4	7.7 ± 2.4	10 mg QD 30 mg QD 60 mg QD	12Weeks	MMDs	MHDs; acute medication use days; ≥50% reduction in MMDs	TEAEs; treatment-related TEAEs; serious TEAEs; treatment-related serious TEAE; nausea; upper respiratory tract infection; nasopharyngitis; constipation; urinary tract infection; fatigue
Pozo-Rosich P et al., 2023	Multinational RCT	ICHD-3	30/225; 42.0± 12.4	66/452; 42.15± 12.10	18.9 ± 4.8	18.9 ± 5.2	60 mg QD 30 mg BID	12Weeks	MMDs	MHDs; acute medication use days; ≥50% reduction in MMDs	TEAEs; treatment-related TEAEs; serious TEAEs; nausea; upper respiratory tract infection; nasopharyngitis; constipation; urinary tract infection; fatigue
Tassorelli C et al., 2024	Multinational RCT	ICHD-3	16/141; 43.4± 10.3	17/139; 40.9± 10.7	9·3 ± 2·4	9·1 ± 2·3	60 mg QD	12Weeks	MMDs	MHDs; acute medication use days; ≥50% reduction in MMDs	TEAEs; treatment-related TEAEs; serious TEAEs; nausea; nasopharyngitis; constipation; urinary tract infection

Data are mean ± SD. RCT, randomized controlled trial; ICHD, the International Classification of Headache Disorders; QD, quaque die, once daily; BID, both in die, twice daily; MMDs, Monthly migraine days; MHDs, Monthly headache days; TEAEs, treatment-emergent adverse events

### Risk of bias and quality of evidence

**Fig. 2:**
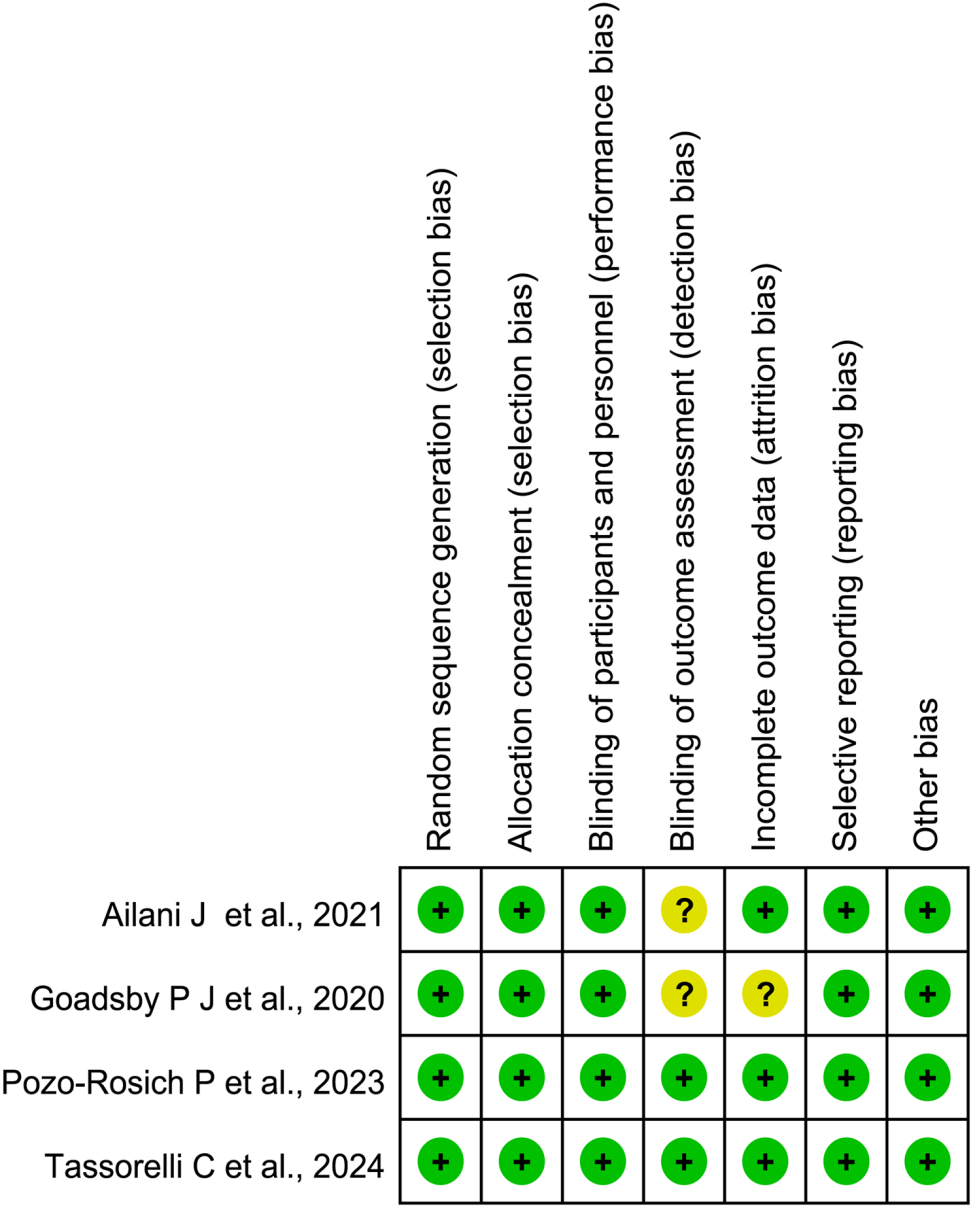
Risk of bias for included trials

**Fig. 3:**
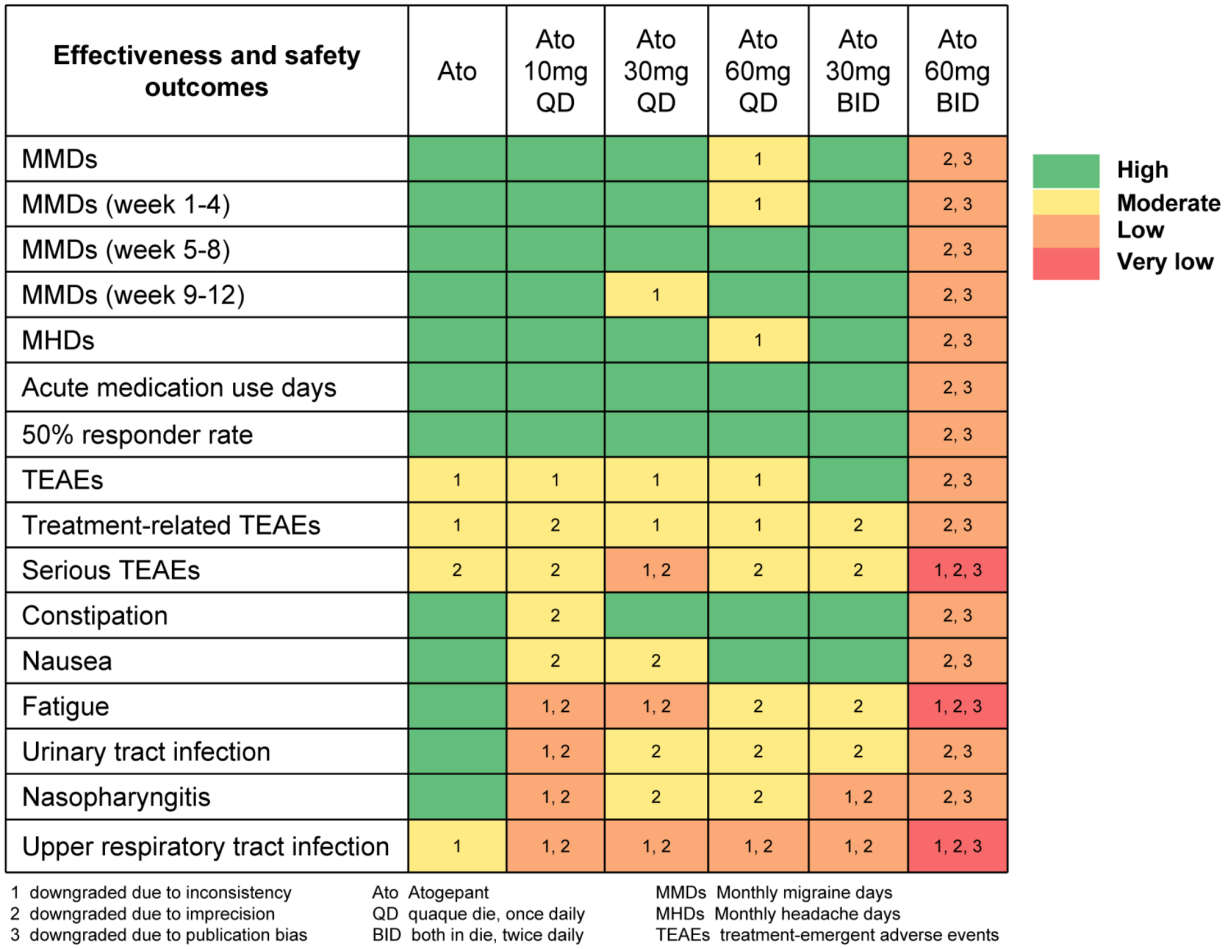
Summary of GRADE results for each outcomes

Figure 2 displays the results from the risk of bias assessment based on the 7-item criteria in RevMan 5.3. Four studies evaluating the efficacy and safety of atogepant for migraine prevention were included [13, 14, 23, 24], all of which were randomized, double-blind, placebo-controlled trials. These RCTs described the randomization procedure in enough detail, and were considered as having a low risk of bias, whereas the risks for detection bias were unclear in two studies [23, 24], and the Goadsby P J et al. 2020 study was also assessed as having a unclear risk. In addition, the quality of the evidence for the effectiveness and safety outcomes was summarized in Fig. 3 and Supplementary Table 1.

### Effectiveness of atogepant for the treatment of migraine prevention

#### The reduction of monthly migraine days (MMDs)

**Fig. 4:**
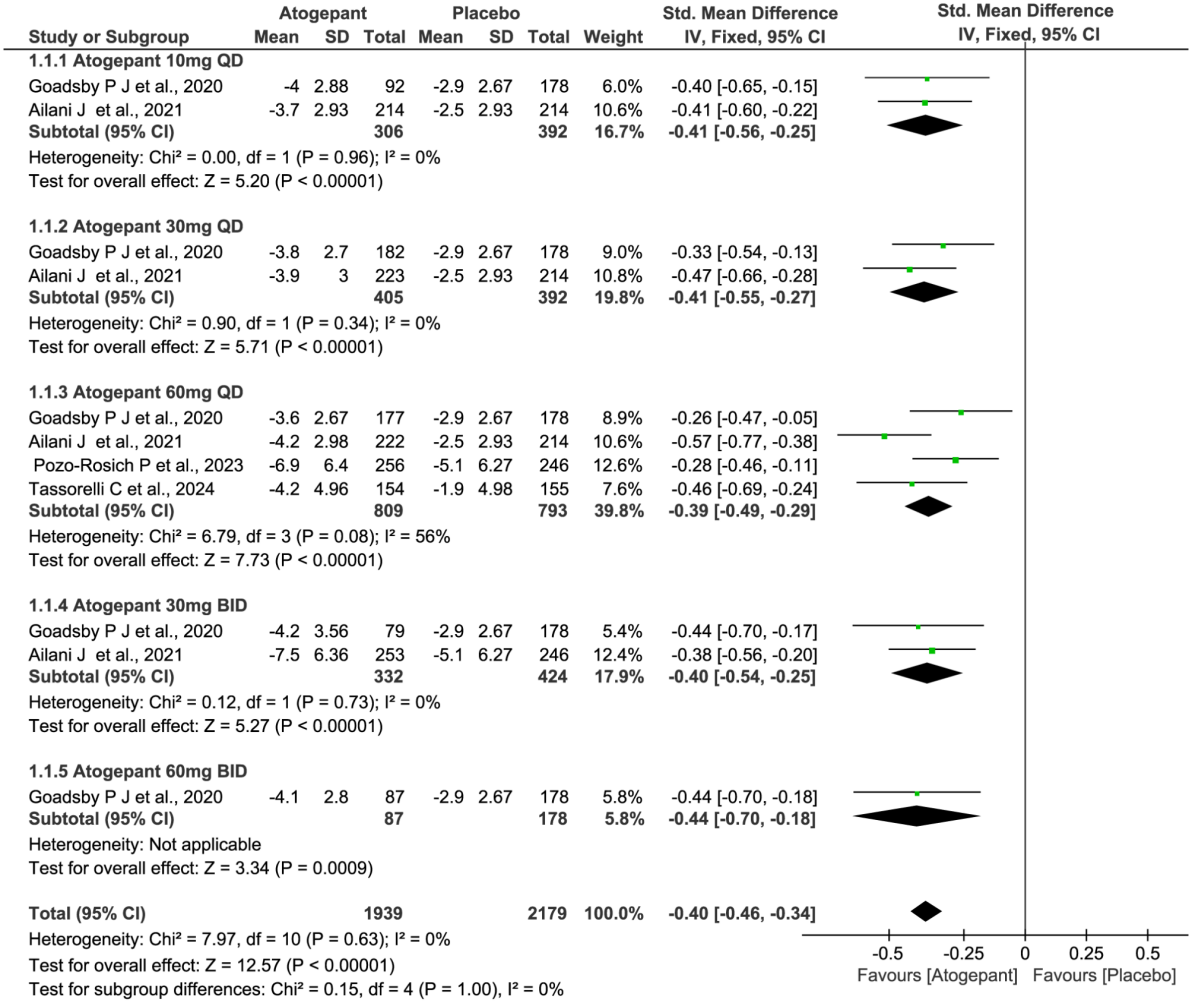
Meta-analysis of the reduction of MMDs in different doses compared with placebo. The diamond indicates the estimated standardized mean difference with 95% confidence interval for the pooled patients. MMDs, Monthly migraine days; M-H, Mantel-Haenszel; CI, confidence interval

**Fig. 5:**
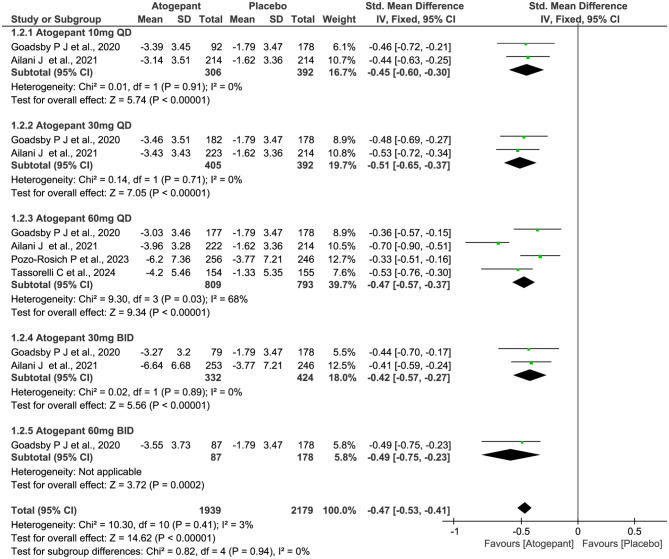
Meta-analysis of the reduction of MMDs in different doses at week 4. The diamond indicates the estimated standardized mean difference with 95% confidence interval for the pooled patients. MMDs, Monthly migraine days; M-H, Mantel-Haenszel; CI, confidence interval

**Fig. 6:**
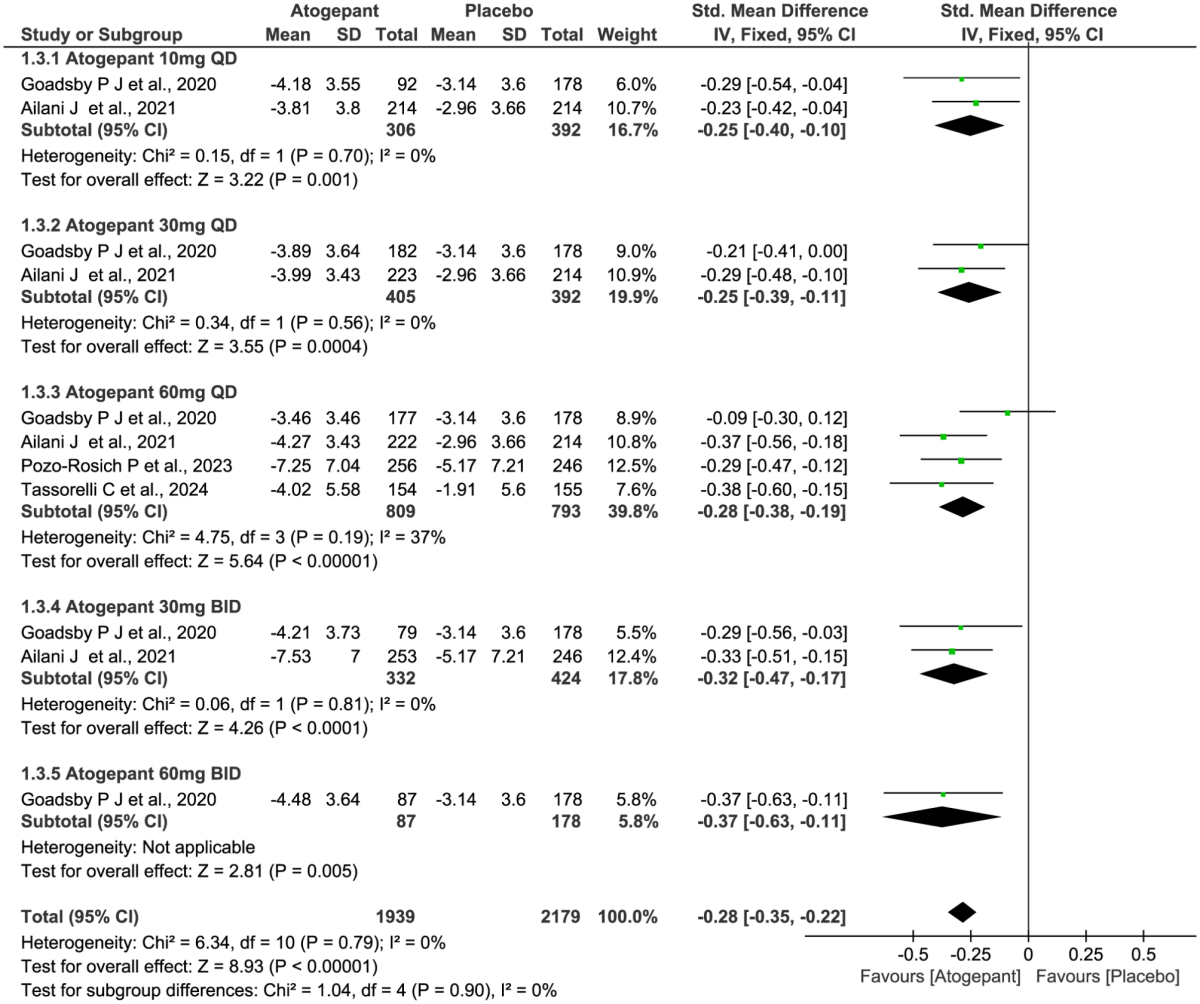
Meta-analysis of the reduction of MMDs in different doses at week 8. The diamond indicates the estimated standardized mean difference with 95% confidence interval for the pooled patients. MMDs, Monthly migraine days; M-H, Mantel-Haenszel; CI, confidence interval

**Fig. 7:**
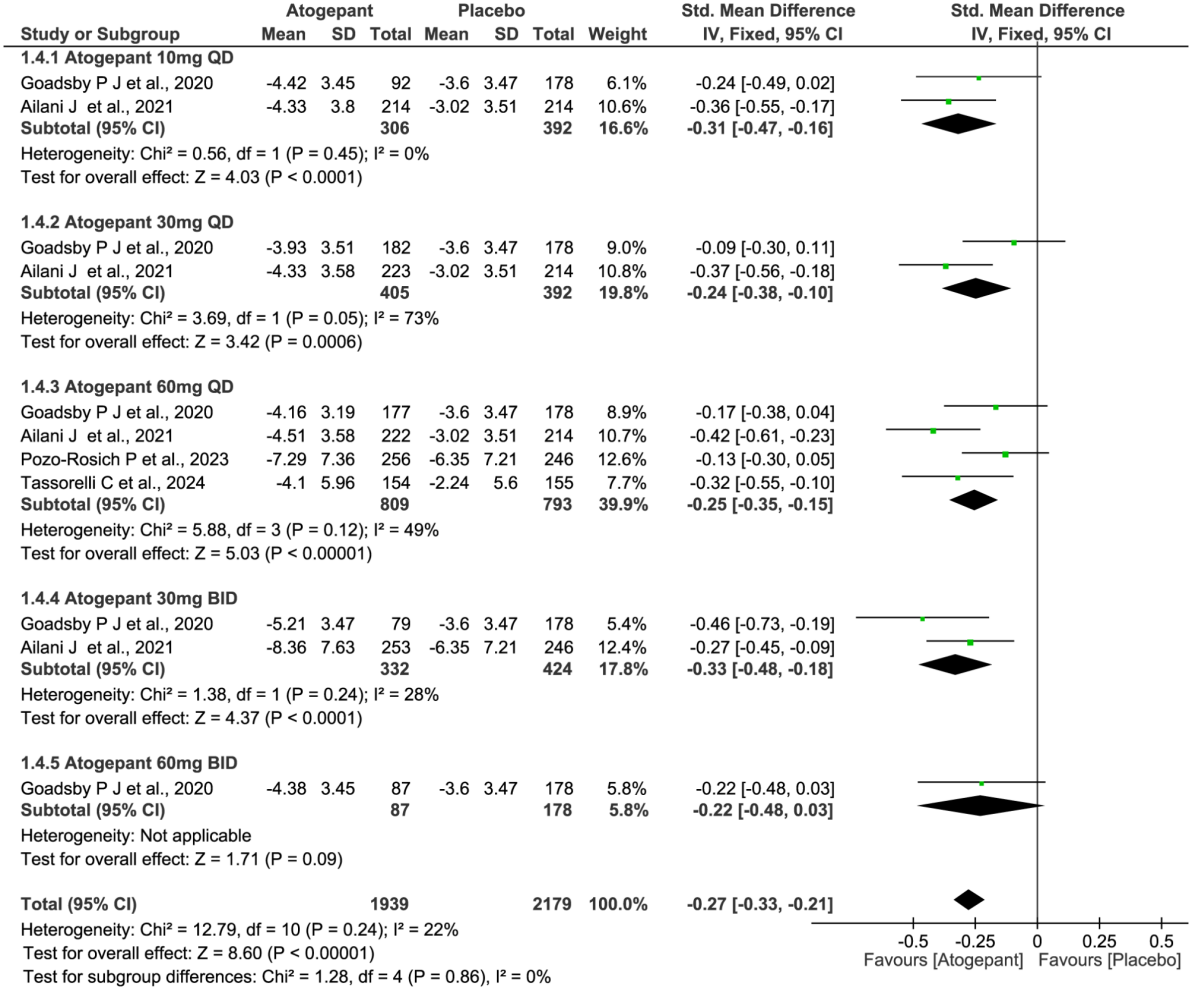
Meta-analysis of the reduction of MMDs in different doses at week 12. The diamond indicates the estimated standardized mean difference with 95% confidence interval for the pooled patients. MMDs, Monthly migraine days; M-H, Mantel-Haenszel; CI, confidence interval

All four trials (2732 subjects) included in this meta-analysis were evaluated for the reduction of MMDs. As shown in Fig. 4, compared with placebo, patients receiving atogepant treatment achieved a significant decrease in the numbers of MMDs (SMD − 0.40, 95% CI -0.46 to -0.34, *P*˂0.00001), with statistically significant differences observed across all dosage groups. However, there were no significant differences among the various dosage groups (*P* = 1.00, *I*^*2*^ = 0%). Furthermore, the results showed similar trends over different time periods from baseline to week 4, 8, and 12 (week 4: SMD − 0.47, 95% CI -0.53 to -0.41, *P*˂0.00001; week 8: SMD − 0.28, 95% CI -0.35 to -0.22, *P*˂0.00001; and week 12: SMD − 0.27, 95% CI -0.33 to -0.21, *P*˂0.00001; Figs. 5, 6 and 7), but notably, greater mean decreases in MMDs were observed with atogepant from baseline to week 4. The total *I*^2^ value (χ^2^ = 7.97, *P* = 0.63, *I*^2^ = 0%) revealed non-significant heterogeneity among the included trials.

### The reduction of monthly headache days (MHDs)

**Fig. 8:**
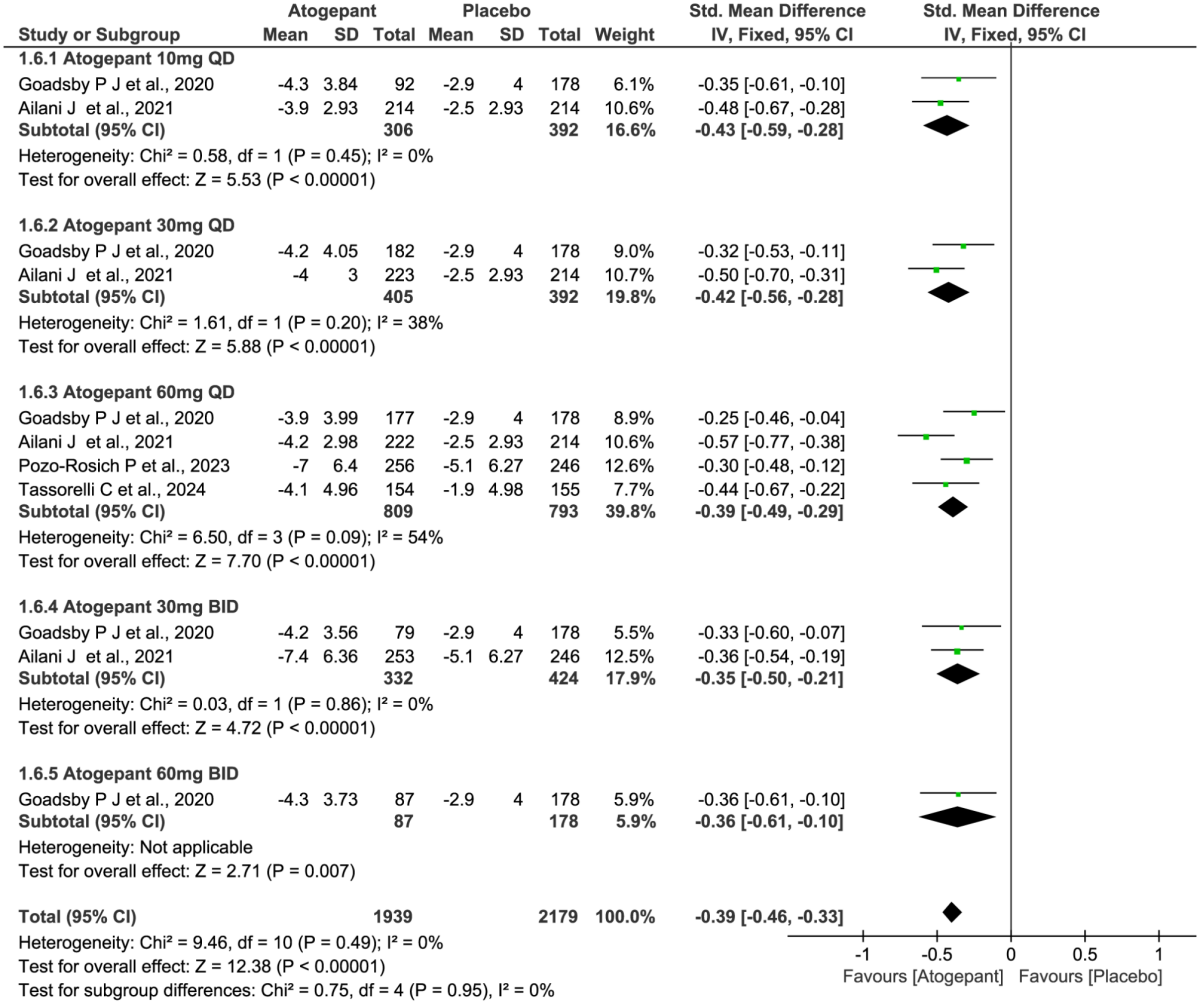
Meta-analysis of the reduction of MHDs in different doses compared with placebo. The diamond indicates the estimated standardized mean difference with 95% confidence interval for the pooled patients. MHDs, Monthly headache days; M-H, Mantel-Haenszel; CI, confidence interval

Four trials of 2732 subjects were included in this meta-analysis and evaluated for the reduction of MHDs. The SMD after treatment favored atogepant over placebo (SMD − 0.39, 95% CI -0.46 to -0.33, *P*˂0.00001; Fig. 8), and the subgroup analysis didn’t show a remarkable difference in the reduction of MHDs from different dosage of atogepant over control therapy (χ^2^ = 0.75, *P* = 0.95, *I*^2^ = 0%). Furthermore, the total(χ^2^ = 9.46, *P* = 0.49, *I*^2^ = 0%) or subgroup *I*^2^ value on the reduction of MHDs revealed a non-significant heterogeneity among the included trials except for the atogepant 60 mg QD group (χ^2^ = 6.50, *P* = 0.09, *I*^2^ = 54%).

### The reduction of acute medication use days

**Fig. 9:**
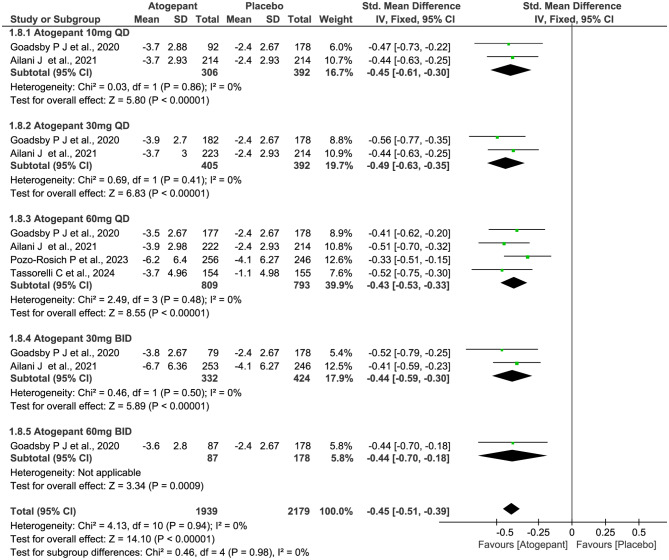
Meta-analysis of the reduction of acute medication use days in different doses compared with placebo. The diamond indicates the estimated standardized mean difference with 95% confidence interval for the pooled patients. M-H, Mantel-Haenszel; CI, confidence interval

Four trials with a total of 2732 subjects included for the outcomes. As shown in Fig. 9, atogepant also showed benefits with over placebo at the reduction of acute medication use days (SMD − 0.45, 95% CI -0.51 to -0.39, *P*˂0.00001), while no dose-related improvements were found in different dosage groups (χ^2^ = 0.46, *P* = 0.98, *I*^2^ = 0%), and continuing to increase the dose of the medication did not further improve the efficacy. The total or subgroup *I*^2^ value revealed non-significant heterogeneity among the included trials.

### 50% responder rate in monthly migraine days

**Fig. 10:**
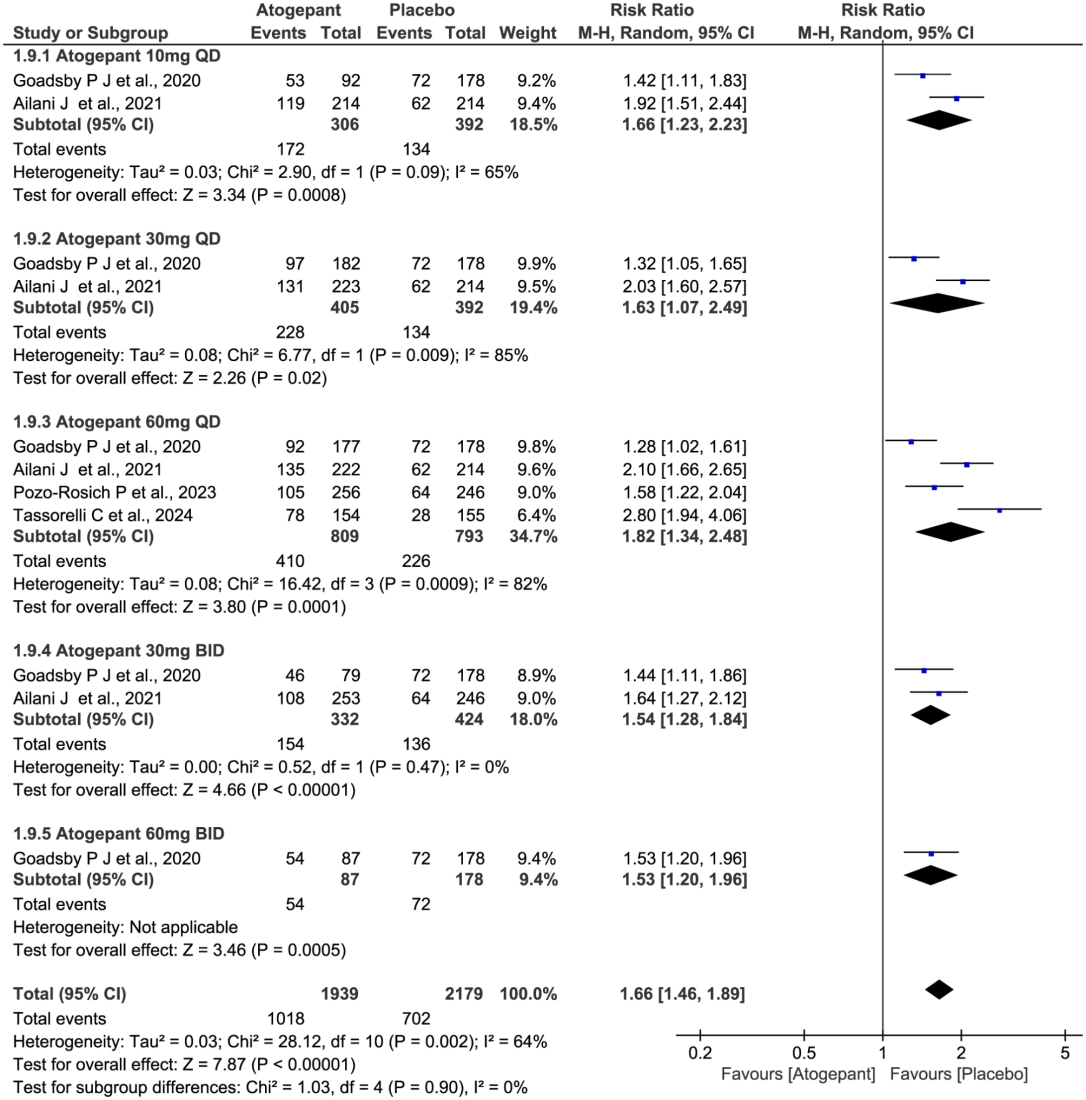
Meta-analysis of the 50% responder rates in diferent doses compared with placebo. The diamond indicates the estimated relative risk with 95% confidence interval for the pooled patients. M-H, Mantel-Haenszel; CI, confidence interval

The 50% reduction rate, when the MMDs were reduced by 50% or more over a period of 12 weeks, was counted in four included trials with a total of 2732 subjects. As shown in Fig. 10, the data showed a significant decrease in 50% responder rates of atogepant compared with placebo (RR 1.66, 95% CI 1.46 to 1.89, *P* = 0.002), and the statistical differences observed across all dosage groups. The total *I*^2^ value revealed a moderate heterogeneity among the included trials (χ^2^ = 28.12, *P* = 0.002, *I*^2^ = 64%), and a high level of heterogeneity in two dosage groups (30 mg QD: χ^2^ = 6.77, *P* = 0.009, *I*^*2*^ = 85%; 60 mg QD: χ^2^ = 16.42, *P* = 0.0009, *I*^*2*^ = 82%).

### Safety of atogepant for the treatment of migraine prevention

Safety outcomes were reported in four trials for adverse events (AEs). As shown in Table 2, the atogepant group and two dosage groups (atogepant 30 mg BID or 60 mg BID) showed a significantly higher risk of any treatment-emergent adverse events (TEAEs) and treatment-related TEAEs (TEAEs: all [RR 1.11, 95% CI 1.02–1.21, *P* = 0.02], atogepant 30 mg BID [RR 1.17, 95% CI 1.02–1.34, *P* = 0.02]; treatment-related TEAEs: atogepant 60 mg BID [RR 1.64, 95% CI 1.02–2.63, *P* = 0.04]). Furthermore, there were remarkable differences between atogepant and placebo in the incidence of constipation, nausea, fatigue and urinary tract infection (constipation: all [RR 2.55, 95% CI 1.91–3.41, *P* < 0.00001], atogepant 30 mg QD [RR 2.14, 95% CI 1.10–4.18, *P* = 0.03], atogepant 60 mg QD [RR 2.74, 95% CI 1.74–4.32, *P* < 0.0001], atogepant 30 mg BID [RR 3.03, 95% CI 1.54–5.95, *P* = 0.001]; nausea: all [RR 2.19, 95% CI 1.67–2.87, *P* < 0.00001], atogepant 60 mg QD [RR 2.63, 95% CI 1.71–4.05, *P* < 0.0001], atogepant 30 mg BID [RR 2.10, 95% CI 1.16–3.78, *P* = 0.01]; fatigue: atogepant 60 mg BID [RR 3.07, 95% CI 1.13–8.35, *P* = 0.03], urinary tract infection: all [RR 1.05, 95% CI 1.05–2.11, *P* = 0.03]), some statistically significant differences between the various dosage groups were detected regarding the aforementioned AEs. In addition, there were no significant differences in the incidence of nasopharyngitis and upper respiratory tract infection, serious TEAEs were reported in 22 of the participants in the atogepant or placebo group. Most of the *I*^2^ value revealed a low heterogeneity among the included studies.

**Table 2:** Comparison of main TEAEs between atogepant and placebo

Outcome or Subgroup	Studies	Participants	Statistical Method	Effect Estimate	*P*	*I* ^*2*^
**TEAEs**	4	4256	RR (M-H, Random, 95% CI)	1.11 [1.02, 1.21]	**0.02**	57%
Atogepant 10 mg QD	2	722	RR (M-H, Random, 95% CI)	1.11 [0.78, 1.56]	0.57	85%
Atogepant 30 mg QD	2	819	RR (M-H, Random, 95% CI)	1.08 [0.79, 1.48]	0.64	85%
Atogepant 60 mg QD	4	1654	RR (M-H, Random, 95% CI)	1.09 [0.93, 1.26]	0.28	65%
Atogepant 30 mg BID	2	784	RR (M-H, Random, 95% CI)	1.17 [1.02, 1.34]	**0.02**	0%
Atogepant 60 mg BID	1	277	RR (M-H, Random, 95% CI)	1.18 [0.94, 1.48]	0.16	/
**Treatment-related TEAEs**	4	4256	RR (M-H, Random, 95% CI)	1.09 [0.98, 1.21]	0.12	51%
Atogepant 10 mg QD	2	722	RR (M-H, Random, 95% CI)	0.95 [0.81, 1.12]	0.53	0%
Atogepant 30 mg QD	2	819	RR (M-H, Random, 95% CI)	1.05 [0.74, 1.48]	0.80	60%
Atogepant 60 mg QD	4	1654	RR (M-H, Random, 95% CI)	1.10 [0.91, 1.33]	0.32	68%
Atogepant 30 mg BID	2	784	RR (M-H, Random, 95% CI)	1.15 [0.99, 1.35]	0.07	0%
Atogepant 60 mg BID	1	277	RR (M-H, Random, 95% CI)	1.64 [1.02, 2.63]	**0.04**	/
**Serious TEAEs**	4	4256	RR (M-H, Fixed, 95% CI)	1.13 [0.64, 2.01]	0.67	0%
Atogepant 10 mg QD	2	722	RR (M-H, Fixed, 95% CI)	1.00 [0.22, 4.54]	1.00	0%
Atogepant 30 mg QD	2	819	RR (M-H, Fixed, 95% CI)	0.56 [0.12, 2.58]	0.45	0%
Atogepant 60 mg QD	4	1654	RR (M-H, Fixed, 95% CI)	1.72 [0.73, 4.08]	0.22	19%
Atogepant 30 mg BID	2	784	RR (M-H, Fixed, 95% CI)	1.01 [0.28, 3.73]	0.98	0%
Atogepant 60 mg BID	1	277	RR (M-H, Fixed, 95% CI)	0.41 [0.02, 8.38]	0.56	/
**Constipation**	4	4256	RR (M-H, Fixed, 95% CI)	2.55 [1.91, 3.41]	**< 0.00001**	0%
Atogepant 10 mg QD	2	722	RR (M-H, Fixed, 95% CI)	1.98 [0.97, 4.04]	0.06	0%
Atogepant 30 mg QD	2	819	RR (M-H, Fixed, 95% CI)	2.14 [1.10, 4.18]	**0.03**	0%
Atogepant 60 mg QD	4	1654	RR (M-H, Fixed, 95% CI)	2.74 [1.74, 4.32]	**< 0.0001**	0%
Atogepant 30 mg BID	2	784	RR (M-H, Fixed, 95% CI)	3.03 [1.54, 5.95]	**0.001**	0%
Atogepant 60 mg BID	1	277	RR (M-H, Fixed, 95% CI)	3.07 [0.89, 10.60]	0.08	/
**Nausea**	4	4256	RR (M-H, Fixed, 95% CI)	2.19 [1.67, 2.87]	**< 0.00001**	0%
Atogepant 10 mg QD	2	722	RR (M-H, Fixed, 95% CI)	1.77 [0.83, 3.78]	0.14	25%
Atogepant 30 mg QD	2	819	RR (M-H, Fixed, 95% CI)	1.77 [0.91, 3.44]	0.09	0%
Atogepant 60 mg QD	4	1654	RR (M-H, Fixed, 95% CI)	2.63 [1.71, 4.05]	**< 0.0001**	0%
Atogepant 30 mg BID	2	784	RR (M-H, Fixed, 95% CI)	2.10 [1.16, 3.78]	**0.01**	0%
Atogepant 60 mg BID	1	277	RR (M-H, Fixed, 95% CI)	2.04 [0.84, 4.97]	0.12	/
**Fatigue**	3	3943	RR (M-H, Fixed, 95% CI)	1.06 [0.75, 1.51]	0.73	0%
Atogepant 10 mg QD	2	722	RR (M-H, Fixed, 95% CI)	0.54 [0.16, 1.81]	0.32	0%
Atogepant 30 mg QD	2	819	RR (M-H, Fixed, 95% CI)	0.71 [0.32, 1.57]	0.39	0%
Atogepant 60 mg QD	3	1341	RR (M-H, Fixed, 95% CI)	1.02 [0.57, 1.84]	0.94	0%
Atogepant 30 mg BID	2	784	RR (M-H, Fixed, 95% CI)	1.27 [0.57, 2.79]	0.56	0%
Atogepant 60 mg BID	1	277	RR (M-H, Fixed, 95% CI)	3.07 [1.13, 8.35]	**0.03**	/
**Urinary tract infection**	4	4256	RR (M-H, Fixed, 95% CI)	1.49 [1.05, 2.11]	**0.03**	0%
Atogepant 10 mg QD	2	722	RR (M-H, Fixed, 95% CI)	0.53 [0.19, 1.46]	0.22	0%
Atogepant 30 mg QD	2	819	RR (M-H, Fixed, 95% CI)	1.57 [0.77, 3.19]	0.21	20%
Atogepant 60 mg QD	4	1654	RR (M-H, Fixed, 95% CI)	1.45 [0.82, 2.57]	0.21	0%
Atogepant 30 mg BID	2	784	RR (M-H, Fixed, 95% CI)	2.54 [0.97, 6.62]	0.06	0%
Atogepant 60 mg BID	1	277	RR (M-H, Fixed, 95% CI)	3.07 [0.89, 10.60]	0.08	/
**Nasopharyngitis**	4	4256	RR (M-H, Fixed, 95% CI)	1.12 [0.82, 1.52]	0.47	10%
Atogepant 10 mg QD	2	722	RR (M-H, Fixed, 95% CI)	0.75 [0.31, 1.84]	0.53	22%
Atogepant 30 mg QD	2	819	RR (M-H, Fixed, 95% CI)	1.57 [0.77, 3.19]	0.21	49%
Atogepant 60 mg QD	4	1654	RR (M-H, Fixed, 95% CI)	1.16 [0.74, 1.80]	0.52	48%
Atogepant 30 mg BID	2	784	RR (M-H, Fixed, 95% CI)	0.83 [0.38, 1.82]	0.65	0%
Atogepant 60 mg BID	1	277	RR (M-H, Fixed, 95% CI)	1.53 [0.35, 6.71]	0.57	/
**Upper respiratory tract infection**	3	3943	RR (M-H, Fixed, 95% CI)	0.86 [0.65, 1.13]	0.28	0%
Atogepant 10 mg QD	2	722	RR (M-H, Fixed, 95% CI)	0.85 [0.45, 1.61]	0.62	0%
Atogepant 30 mg QD	2	819	RR (M-H, Fixed, 95% CI)	1.08 [0.64, 1.82]	0.78	0%
Atogepant 60 mg QD	3	1341	RR (M-H, Fixed, 95% CI)	0.67 [0.39, 1.14]	0.14	0%
Atogepant 30 mg BID	2	784	RR (M-H, Fixed, 95% CI)	0.91 [0.45, 1.85]	0.80	0%
Atogepant 60 mg BID	1	277	RR (M-H, Fixed, 95% CI)	0.82 [0.33, 2.04]	0.67	/

TEAEs, treatment-emergent adverse events; QD, quaque die, once daily; BID, both in die, twice daily; RR, relative risk; M-H, Mantel-Haenszel; CI, confidence interval

## Discussion

The current study aimed to evaluate the efficacy and safety of the different-dosage atogepant on patients with migraine through meta-analysis in the phase II RCTs published from 2020 to Feb 2024 [13, 14, 23, 24]. An additional 2 studies were included with 1064 patients on the basis of previous meta-analysis. The overall trend in these results is in accordance with previous studies [11, 12] demonstrating that atogepant for the treatment of migraine prevention were associated with improvements in impairments attributed to migraine and also with increased risk of some adverse effects.

The main differences compared to previous studies [11, 12] are reflected in the following two aspects. In the part of effectiveness, although the results were basically consistent with previous results, demonstrating that atogepant was more efficacious than placebo in preventing migraine with respect to the primary (the reduction of MMDs) and secondary outcomes (the reduction of MHDs, the reduction of acute medication use days and 50% responder rate), it was noteworthy that the heterogeneity level of the combined results in reducing MMDs or MHDs for the conventional dosage group (60 mg QD) of atogepant approved by the FDA had been improved in our study, from 79% to 80% in previous studies to 56% or 54% now respectively. This reduction indicated that the previous results had been further validated and strengthened. In addition to the primary efficacy endpoint MMDs were combined and analysed across the 12-week treatment period, MMDs were calculated during weeks 1–4, 5–8, and 9–12 in this meta-analysis. Compared with placebo, greater mean decreases from baseline in mean MMDs were observed with atogepant during the first 4 weeks of treatment. In terms of safety and tolerability, the results differed from the previous trials with no statistical differences between treatments and control for TEAEs. The present results showed the odds of total TEAEs were signifcantly higher with treatments than placebo (RR 1.11, 95% CI 1.02–1.21, *P* = 0.02), particularly atogepant 30 mg BID group with the largest RR (1.17, 95% CI 1.02–1.34). However, in contrast to our findings, previous results did not demonstrate a significant difference between atogepant and placebo in this regard. The most common TEAEs in the atogepant groups involved gastrointestinal symptoms such as nausea and constipation, which might be related to the blockade of the CGRP receptors in the gastrointestinal system [27, 28]. Furthermore, these potential risk were likely dose-dependent with no statistical differences. In addition, larger dosage (atogepant 60 mg BID) may be linked to increased risk of treatment-related TEAEs (RR 1.64, 95% CI 1.02–2.63, *P* = 0.04; supplementary Fig. 1), such as fatigue (RR 3.07, 95% CI 1.13–8.35, *P* = 0.03; supplementary Fig. 2). And more significantly, the current results showed the risk of treatment-related TEAEs and fatigue in the atogepant 60 mg BID group was markedly higher than in the 10 mg QD group (treatment-related TEAEs: χ^2^ = 4.52, *P* = 0.03, *I*^*2*^ = 77.9%; fatigue: χ^2^ = 4.70, *P* = 0.03, *I*^*2*^ = 78.7%; supplementary Figs. 1 and 2). Furthermore, although there was no statistically significant difference compared with placebo, the risk of urinary tract infection appeared to be potentially higher in the atogepant 30 mg BID or 60 mg BID groups compared to the 10 mg QD group (30 mg BID vs. 10 mg QD: χ^2^ = 4.83, *P* = 0.03, *I*^*2*^ = 79.3%; 60 mg BID vs. 10 mg QD: χ^2^ = 4.60, *P* = 0.03, *I*^*2*^ = 78.3%; supplementary Figs. 3 and 4). However, these findings had not been previously presented or mentioned in systematic reviews. Therefore, given the scarcity of available data, further safety studies are urgently needed to comprehensively assess this potential risk. Furthermore, in consideration of safety concerns, it is advisable and highly recommended to prescribe the lowest effective dosage of atogepant for migraine patients. Other findings were similar to those in a previous study that found the incidence of other common TEAEs including upper respiratory tract infection and nasopharyngitis were comparable as between the active and placebo arms. Serious TEAEs were rare, 2 or 3 participants were reported in any treatment group, but such cases were also balanced between the treatments and placebo.

For the long-term efficacy and safety of atogepant, a phase III, multicenter, randomized, open-label study (NCT03700320) involved patients who had completed the phase 2b/3 trial (NCT02848326) or were newly enrolled in the current trial [29], and received atogepant 60 mg once daily or oral standard care migraine preventive medication. Similarly, the efficacy results were consistent with the previous studies, which showed a benefit of atogepant for reducing in MMDs and suggested an increase in efficacy with the duration of treatment. Of note, TEAEs over time generally increased to 67.0% with no other trends with regard to serious AEs reported during treatment with atogepant for up to 1 year, including the hepatic safety issues from the first-generation small molecule CGRP receptor antagonists involved. Despite the most frequently reported TEAEs were associated with the gastrointestinal system, there were no serious accidents or injuries resulting from a gastrointestinal AE during long-term treatment. Furthermore, compared to the combined results in this meta-analysis, the incidence of gastrointestinal AEs including nausea and constipation was slightly decreased to 6.3% and 7.2% respectively over 52 weeks. Nevertheless, what is noticeable is that long-term medication seems to increase the risk of upper respiratory tract (10.3%) or urinary tract (5.2%) infection, this warrants further safety study given the limited existing data.

### Limitations

Compared with previous studies aimed to assess the efficacy and safety of atogepant in the preventive treatment of migraine, we conducted this meta-analysis based on a more comprehensive study included. Nevertheless, some potential limitations of this study should be necessary to consider. Firstly, the number of included studies was relatively small, only 4 trials with a total of 2813 subjects were included in our analysis so far. Consequently, the funnel plots were not performed to predict publication bias of the meta-analysis due to the small number of RCTs. Secondly, The literature included in different dosage groups except in patients with atogepant 60 mg QD are relatively few, future studies are needed to confirm our results, especially concerning the discrepancy among different dose groups in efficacy and safety for migraine prophylaxis. Thirdly, this meta-analysis only focused on the short-term pain responses and side effects of atogepant during the 12-week period and neglected the long-term efficacy and safety due to the limited data. In addition to the study by Ashina M et al. [29], so far there are two studies are currently evaluating the long-term safety and tolerability of atogepant in adult patients with episodic or chronic migraine (NCT04686136 and NCT04437433), long-term efficacy and safety can be conducted when results of on-going studies above become available. Finally, several types of biases may limit the validity of the overall findings in this meta-analysis, such as some potential bias originated from inconsistencies in the patient characteristics in both the episodic and chronic migraine, and these data may not be entirely generalizable to real-world practice.

## Conclusions

This systematic review and meta-analysis compared different doses of atogepant with placebo for 12 weeks, and suggested that atogepant are effective and tolerable in a non-dose-dependent manner for migraine prophylaxis, furthermore, greater mean decreases from baseline in mean MMDs were observed with atogepant during the first 4 weeks of treatment. However, AEs included nausea, constipation are common, and the risk of upper respiratory tract and urinary tract infection is also notable over time generally increased. Besides, larger dosage of atogepant may be linked to increased risk of treatment-related TEAEs such as fatigue. Consequently, it is critical to weigh the benefits of different doses against the risk of AEs in clinical application of atogepant. Longer and multi-dose trials with larger sample sizes are needed to determine the efficacy and safety of atogepant for migraine prevention in the future.

### Electronic supplementary material

Below is the link to the electronic supplementary material.

Supplementary Material 1

Supplementary Material 2

Supplementary Material 3

## Data Availability

Data have been provided within the manuscript or additional file 1, 2,and 3.

## Abbreviations

CGRP: The Calcitonin Gene-Related Peptide
mAbs: Monoclonal Antibodies
ICHD: The International Classification of Headache Disorders
QD: Quaque Die, Once Daily
BID: Both In Die, Twice Daily
RR: Relative Risk
SMD: Standardized Mean Difference
CI: Confidence Intervals
M-H: Mantel-Haenszel
MMDs: The Reduction Of Monthly Migraine Days
MHDs: The Reduction Of Monthly Headache Days
AEs: Adverse Events
TEAEs: Treatment-Emergent Adverse Events

## References

[1] Munjal S, Singh P, Reed ML et al (2020) Most bothersome symptom in persons with migraine: results from the Migraine in America symptoms and treatment (MAST) study. Headache 60:416–429 31837007 10.1111/head.13708 PMC7027490

[2] Dereje N (2020) Global burden of 369 diseases and injuries in 204 countries and territories, 1990–2019: a systematic analysis for the global burden of Disease Study 2019. Lancet 396:1204–1222 33069326 10.1016/S0140-6736(20)30925-9 PMC7567026

[3] Steiner TJ, Stovner LJ, Jensen R et al (2020) Migraine remains second among the world’s causes of disability, and first among young women: findings from GBD2019. J Headache Pain 21:137 33267788 10.1186/s10194-020-01208-0 PMC7708887

[4] Ailani J, Burch RC, Robbins MS (2021) The American Headache Society Consensus Statement: update on integrating new migraine treatments into clinical practice. Headache 61:1021–1039 34160823 10.1111/head.14153

[5] Blumenfeld AM, Bloudek LM, Becker WJ et al (2013) Patterns of use and reasons for discontinuation of prophylactic medications for episodic migraine and chronic migraine: results from the second international burden of migraine study (IBMS-II). Headache 53:644–655 23458496 10.1111/head.12055

[6] Ojo A, Zhang S, Bleibdrey N et al (2023) Persistence and switching patterns of migraine prophylactic medications in Canada: a retrospective claims analysis comparing adherence and evaluating the economic burden of illness. J Pharm Pharm Sciences: Publication Can Soc Pharm Sci Societe canadienne des Sci Pharmaceutiques 25:402–417 10.18433/jpps33158 36623477

[7] Hepp Z, Bloudek LM, Varon SF (2014) Systematic review of Migraine Prophylaxis Adherence and Persistence. J Managed care Pharmacy: JMCP 20:22–33 10.18553/jmcp.2014.20.1.22 PMC10437641 24372457

[8] Irimia P, García-Azorín D, Núez M et al (2022) Persistence, use of resources and costs in patients under migraine preventive treatment: the PERSEC study. J Headache Pain 23:1–15 35794535 10.1186/s10194-022-01448-2 PMC9261063

[9] Jinesh S (2023) Pharmaceutical aspects of novel CGRP inhibitors used in the prophylaxis and treatment of migraine. Inflammopharmacology 31:2245–2251 37421480 10.1007/s10787-023-01276-z

[10] Tso AR, Goadsby PJ (2017) Anti-CGRP monoclonal antibodies: the next era of Migraine Prevention? Curr Treat Options Neurol 19:27 28653227 10.1007/s11940-017-0463-4 PMC5486583

[11] Tao X, Yan Z, Meng J et al (2022) The efficacy and safety of atogepant for the prophylactic treatment of migraine: evidence from randomized controlled trials. J Headache Pain 23:19 35093013 10.1186/s10194-022-01391-2 PMC8903713

[12] Lattanzi S, Trinka E, Altamura C et al (2022) Atogepant for the Prevention of episodic migraine in adults: a systematic review and Meta-analysis of efficacy and safety. Neurol Therapy 11:1235–1252 10.1007/s40120-022-00370-8 PMC9338214 35705886

[13] Pozo-Rosich P, Ailani J, Ashina M et al (2023) Atogepant for the preventive treatment of chronic migraine (PROGRESS): a randomised, double-blind, placebo-controlled, phase 3 trial. Lancet 402:775–785 37516125 10.1016/S0140-6736(23)01049-8

[14] Tassorelli C, Nagy K, Pozo-Rosich P et al (2024) Safety and efficacy of atogepant for the preventive treatment of episodic migraine in adults for whom conventional oral preventive treatments have failed (ELEVATE): a randomised, placebo-controlled, phase 3b trial. Lancet Neurol 23:382–392 38364831 10.1016/S1474-4422(24)00025-5

[15] Higgins JPT, Altman DG, Gtzsche PC et al (2011) The Cochrane collaboration’s tool for assessing risk of bias in randomised trials. BMJ 343:d5928 22008217 10.1136/bmj.d5928 PMC3196245

[16] Higgins JPT, Altman DG (2011) Assessing risk of Bias in included studies, Cochrane Handbook for systematic reviews of interventions: Cochrane Book Series. John Wiley & Sons, Ltd.

[17] Mascarenhas RO, Souza MB, Oliveira MX et al (2021) Association of therapies with reduced Pain and Improved Quality of Life in patients with Fibromyalgia: a systematic review and Meta-analysis. JAMA Intern Med 181:104–112 33104162 10.1001/jamainternmed.2020.5651 PMC7589080

[18] Balshem H, Helfand M, Schünemann HJ et al (2011) GRADE guidelines: 3. Rating the quality of evidence. J Clin Epidemiol 64:401–406 21208779 10.1016/j.jclinepi.2010.07.015

[19] Higgins J, Thompson SG (2002) Quantifying heterogeneity in a meta-analysis. Stat Med 21:1539–1558 12111919 10.1002/sim.1186

[20] Hou M, Xing H, Li C et al (2020) Short-term efficacy and safety of lasmiditan, a novel 5-HT1F receptor agonist, for the acute treatment of migraine: a systematic review and meta-analysis. J Headache Pain 21:66 32503415 10.1186/s10194-020-01138-x PMC7275414

[21] Hou M, Xing H, Cai Y et al (2017) The effect and safety of monoclonal antibodies to calcitonin gene-related peptide and its receptor on migraine: a systematic review and meta-analysis. J Headache Pain 18:42 28389966 10.1186/s10194-017-0750-1 PMC5383797

[22] Tufanaru C, Munn Z, Stephenson M et al (2015) Fixed or random effects meta-analysis? Common methodological issues in systematic reviews of effectiveness. Int J Evid Based Healthc 13:196–207 26355603 10.1097/XEB.0000000000000065

[23] Goadsby PJ, Dodick DW, Ailani J et al (2020) Safety, tolerability, and efficacy of orally administered atogepant for the prevention of episodic migraine in adults: a double-blind, randomised phase 2b/3 trial. Lancet Neurol 19:727–737 32822633 10.1016/S1474-4422(20)30234-9

[24] Ailani J, Lipton RB, Goadsby PJ et al (2021) Atogepant for the Preventive treatment of Migraine. N Engl J Med 385:695–706 34407343 10.1056/NEJMoa2035908

[25] Headache Classification Committee of the International Headache Society IHS (2013) The International classification of Headache disorders, 3rd edition (beta version). Cephalalgia 33:629–808 23771276 10.1177/0333102413485658

[26] Headache Classification Committee of the International Headache Society (IHS) (2018) The International Classification of Headache Disorders, 3rd edition. *Cephalalgia* 38:1-211 10.1177/0333102417738202 29368949

[27] Haanes KA, Edvinsson L, Sams A (2020) Understanding side-effects of anti-CGRP and anti-CGRP receptor antibodies. J Headache Pain 21:26 32178623 10.1186/s10194-020-01097-3 PMC7077114

[28] Ailani J, Kaiser EA, Mathew PG et al (2022) Role of calcitonin gene-related peptide on the gastrointestinal symptoms of migraine-clinical considerations: a narrative review. Neurology 99:841–853 36127137 10.1212/WNL.0000000000201332 PMC9651456

[29] Ashina M, Tepper SJ, Reuter U et al (2023) Once-daily oral atogepant for the long-term preventive treatment of migraine: findings from a multicenter, randomized, open-label, phase 3 trial. Headache 63:79–88 36651532 10.1111/head.14439 PMC10107835

