# Supplementary material for: Efficacy and safety of atogepant, a small molecule CGRP receptor antagonist, for the preventive treatment of migraine: a systematic review and meta-analysis
Source: J Headache Pain. 2024 Jul 19;25(1):116. doi: 10.1186/s10194-024-01822-2 (PMC11264921; doi:10.1186/s10194-024-01822-2)
Supplement: Supplementary file 1 — Supplementary Material 1 [file 10194_2024_1822_MOESM1_ESM.doc]

**Search strategy**

Medical Subject Headings [MeSH] and text word [tw] terms were used in typing different keywords using Boolean operator “OR”. Boolean operator “AND” was used to combine all search histories at the completion of interrogation of the respective databases.

Taking the PubMed database as an example.

#1

"Migraine Disorders"[Mesh]

#2

((((((((((((((((((((((((((((((((("Disorder, Migraine"[Title/Abstract]) OR ("Disorders, Migraine"[Title/Abstract])) OR ("Migraine Disorder"[Title/Abstract])) OR ("Headache, Migraine"[Title/Abstract])) OR ("Headaches, Migraine"[Title/Abstract])) OR ("Migraine Headaches"[Title/Abstract])) OR (Migraine[Title/Abstract])) OR (Migraines[Title/Abstract])) OR ("Migraine Headache"[Title/Abstract])) OR ("Acute Confusional Migraine"[Title/Abstract])) OR ("Acute Confusional Migraines"[Title/Abstract])) OR ("Migraine, Acute Confusional"[Title/Abstract])) OR ("Migraines, Acute Confusional"[Title/Abstract])) OR ("Status Migrainosus"[Title/Abstract])) OR ("Abdominal Migraine"[Title/Abstract])) OR ("Abdominal Migraines"[Title/Abstract])) OR ("Migraine, Abdominal"[Title/Abstract])) OR ("Migraines, Abdominal"[Title/Abstract])) OR ("Cervical Migraine Syndrome"[Title/Abstract])) OR ("Cervical Migraine Syndromes"[Title/Abstract])) OR ("Migraine Syndrome, Cervical"[Title/Abstract])) OR ("Migraine Syndromes, Cervical"[Title/Abstract])) OR ("Hemicrania Migraine"[Title/Abstract])) OR ("Hemicrania Migraines"[Title/Abstract])) OR ("Migraine, Hemicrania"[Title/Abstract])) OR ("Migraines, Hemicrania"[Title/Abstract])) OR ("Migraine Variant"[Title/Abstract])) OR ("Migraine Variants"[Title/Abstract])) OR ("Variant, Migraine"[Title/Abstract])) OR ("Variants, Migraine"[Title/Abstract])) OR ("Sick Headache"[Title/Abstract])) OR ("Headache, Sick"[Title/Abstract])) OR ("Headaches, Sick"[Title/Abstract])) OR ("Sick Headaches"[Title/Abstract])

#3: #1 OR #2

("Migraine Disorders"[Mesh]) OR (((((((((((((((((((((((((((((((((("Disorder, Migraine"[Title/Abstract]) OR ("Disorders, Migraine"[Title/Abstract])) OR ("Migraine Disorder"[Title/Abstract])) OR ("Headache, Migraine"[Title/Abstract])) OR ("Headaches, Migraine"[Title/Abstract])) OR ("Migraine Headaches"[Title/Abstract])) OR (Migraine[Title/Abstract])) OR (Migraines[Title/Abstract])) OR ("Migraine Headache"[Title/Abstract])) OR ("Acute Confusional Migraine"[Title/Abstract])) OR ("Acute Confusional Migraines"[Title/Abstract])) OR ("Migraine, Acute Confusional"[Title/Abstract])) OR ("Migraines, Acute Confusional"[Title/Abstract])) OR ("Status Migrainosus"[Title/Abstract])) OR ("Abdominal Migraine"[Title/Abstract])) OR ("Abdominal Migraines"[Title/Abstract])) OR ("Migraine, Abdominal"[Title/Abstract])) OR ("Migraines, Abdominal"[Title/Abstract])) OR ("Cervical Migraine Syndrome"[Title/Abstract])) OR ("Cervical Migraine Syndromes"[Title/Abstract])) OR ("Migraine Syndrome, Cervical"[Title/Abstract])) OR ("Migraine Syndromes, Cervical"[Title/Abstract])) OR ("Hemicrania Migraine"[Title/Abstract])) OR ("Hemicrania Migraines"[Title/Abstract])) OR ("Migraine, Hemicrania"[Title/Abstract])) OR ("Migraines, Hemicrania"[Title/Abstract])) OR ("Migraine Variant"[Title/Abstract])) OR ("Migraine Variants"[Title/Abstract])) OR ("Variant, Migraine"[Title/Abstract])) OR ("Variants, Migraine"[Title/Abstract])) OR ("Sick Headache"[Title/Abstract])) OR ("Headache, Sick"[Title/Abstract])) OR ("Headaches, Sick"[Title/Abstract])) OR ("Sick Headaches"[Title/Abstract]))

#4

"Calcitonin Gene-Related Peptide Receptor Antagonists"[Mesh]

#5

(((((((((((((((((("Calcitonin Gene Related Peptide Receptor Antagonists"[Title/Abstract]) OR ("CGRP-R Inhibitors"[Title/Abstract])) OR ("CGRP R Inhibitors"[Title/Abstract])) OR ("Inhibitors, CGRP-R"[Title/Abstract])) OR ("CGRP-R Inhibitor"[Title/Abstract])) OR ("CGRP R Inhibitor"[Title/Abstract])) OR ("Inhibitor, CGRP-R"[Title/Abstract])) OR ("Calcitonin Gene-Related Peptide Receptor Antagonist"[Title/Abstract])) OR ("Calcitonin Gene Related Peptide Receptor Antagonist"[Title/Abstract])) OR ("CGRP Receptor Antagonist"[Title/Abstract])) OR ("Antagonist, CGRP Receptor"[Title/Abstract])) OR ("Receptor Antagonist, CGRP"[Title/Abstract])) OR ("CGRP Receptor Antagonists"[Title/Abstract])) OR ("Antagonists, CGRP Receptor"[Title/Abstract])) OR ("Receptor Antagonists, CGRP"[Title/Abstract])) OR (GEPANTS[Title/Abstract])) OR (GEPANT[Title/Abstract])) OR (atogepant[Title/Abstract])) OR (AGN-241689[Title/Abstract])

#6: #4 OR #5

("Calcitonin Gene-Related Peptide Receptor Antagonists"[Mesh]) OR ((((((((((((((((((("Calcitonin Gene Related Peptide Receptor Antagonists"[Title/Abstract]) OR ("CGRP-R Inhibitors"[Title/Abstract])) OR ("CGRP R Inhibitors"[Title/Abstract])) OR ("Inhibitors, CGRP-R"[Title/Abstract])) OR ("CGRP-R Inhibitor"[Title/Abstract])) OR ("CGRP R Inhibitor"[Title/Abstract])) OR ("Inhibitor, CGRP-R"[Title/Abstract])) OR ("Calcitonin Gene-Related Peptide Receptor Antagonist"[Title/Abstract])) OR ("Calcitonin Gene Related Peptide Receptor Antagonist"[Title/Abstract])) OR ("CGRP Receptor Antagonist"[Title/Abstract])) OR ("Antagonist, CGRP Receptor"[Title/Abstract])) OR ("Receptor Antagonist, CGRP"[Title/Abstract])) OR ("CGRP Receptor Antagonists"[Title/Abstract])) OR ("Antagonists, CGRP Receptor"[Title/Abstract])) OR ("Receptor Antagonists, CGRP"[Title/Abstract])) OR (GEPANTS[Title/Abstract])) OR (GEPANT[Title/Abstract])) OR (atogepant[Title/Abstract])) OR (AGN-241689[Title/Abstract]))

#7

((("Randomized Controlled Trials"[Title/Abstract]) OR (RCT[Title/Abstract])) OR (Randomly[Title/Abstract])) OR (random[Title/Abstract])

#8: #3 AND #6 AND #7

(((("Migraine Disorders"[Mesh]) OR (((((((((((((((((((((((((((((((((("Disorder, Migraine"[Title/Abstract]) OR ("Disorders, Migraine"[Title/Abstract])) OR ("Migraine Disorder"[Title/Abstract])) OR ("Headache, Migraine"[Title/Abstract])) OR ("Headaches, Migraine"[Title/Abstract])) OR ("Migraine Headaches"[Title/Abstract])) OR (Migraine[Title/Abstract])) OR (Migraines[Title/Abstract])) OR ("Migraine Headache"[Title/Abstract])) OR ("Acute Confusional Migraine"[Title/Abstract])) OR ("Acute Confusional Migraines"[Title/Abstract])) OR ("Migraine, Acute Confusional"[Title/Abstract])) OR ("Migraines, Acute Confusional"[Title/Abstract])) OR ("Status Migrainosus"[Title/Abstract])) OR ("Abdominal Migraine"[Title/Abstract])) OR ("Abdominal Migraines"[Title/Abstract])) OR ("Migraine, Abdominal"[Title/Abstract])) OR ("Migraines, Abdominal"[Title/Abstract])) OR ("Cervical Migraine Syndrome"[Title/Abstract])) OR ("Cervical Migraine Syndromes"[Title/Abstract])) OR ("Migraine Syndrome, Cervical"[Title/Abstract])) OR ("Migraine Syndromes, Cervical"[Title/Abstract])) OR ("Hemicrania Migraine"[Title/Abstract])) OR ("Hemicrania Migraines"[Title/Abstract])) OR ("Migraine, Hemicrania"[Title/Abstract])) OR ("Migraines, Hemicrania"[Title/Abstract])) OR ("Migraine Variant"[Title/Abstract])) OR ("Migraine Variants"[Title/Abstract])) OR ("Variant, Migraine"[Title/Abstract])) OR ("Variants, Migraine"[Title/Abstract])) OR ("Sick Headache"[Title/Abstract])) OR ("Headache, Sick"[Title/Abstract])) OR ("Headaches, Sick"[Title/Abstract])) OR ("Sick Headaches"[Title/Abstract]))) AND (("Calcitonin Gene-Related Peptide Receptor Antagonists"[Mesh]) OR ((((((((((((((((((("Calcitonin Gene Related Peptide Receptor Antagonists"[Title/Abstract]) OR ("CGRP-R Inhibitors"[Title/Abstract])) OR ("CGRP R Inhibitors"[Title/Abstract])) OR ("Inhibitors, CGRP-R"[Title/Abstract])) OR ("CGRP-R Inhibitor"[Title/Abstract])) OR ("CGRP R Inhibitor"[Title/Abstract])) OR ("Inhibitor, CGRP-R"[Title/Abstract])) OR ("Calcitonin Gene-Related Peptide Receptor Antagonist"[Title/Abstract])) OR ("Calcitonin Gene Related Peptide Receptor Antagonist"[Title/Abstract])) OR ("CGRP Receptor Antagonist"[Title/Abstract])) OR ("Antagonist, CGRP Receptor"[Title/Abstract])) OR ("Receptor Antagonist, CGRP"[Title/Abstract])) OR ("CGRP Receptor Antagonists"[Title/Abstract])) OR ("Antagonists, CGRP Receptor"[Title/Abstract])) OR ("Receptor Antagonists, CGRP"[Title/Abstract])) OR (GEPANTS[Title/Abstract])) OR (GEPANT[Title/Abstract])) OR (atogepant[Title/Abstract])) OR (AGN-241689[Title/Abstract]))))) AND ((((("Randomized Controlled Trials"[Title/Abstract]) OR (RCT[Title/Abstract])) OR (Randomly[Title/Abstract])) OR (random[Title/Abstract]))
