# Supplementary material for: Efficacy and safety of atogepant, a small molecule CGRP receptor antagonist, for the preventive treatment of migraine: a systematic review and meta-analysis
Source: J Headache Pain. 2024 Jul 19;25(1):116. doi: 10.1186/s10194-024-01822-2 (PMC11264921; doi:10.1186/s10194-024-01822-2)
Supplement: Supplementary file 2 — Supplementary Material 2 [file 10194_2024_1822_MOESM2_ESM.doc]

The Grading **of Recommendations Assessment, Development and Evaluation (GRADE) approach for rating the quality of estimates of treatment effect size. The specific evaluation criteria are as follows：**

**(A) Study design:** All of the included studies were randomized, double-blind, placebo-controlled trials. In general, RCTs will provide high quality evidence if the design (risk of bias) does not have important limitations.

**(B) Inconsistency:** Judging the extent of heterogeneity by *I^2^* value, and in general, inconsistency of results when the *I^2^* statistic was greater than 50% or when pooling was not possible. Simultaneously, it is also crucial to take into account other factors that may potentially influence the consistency of the results.

**(C) Indirectness:** Evaluating indirectness of the target population, intervention and outcomes. In this meta-analysis, these were assured by limiting the included outcomes specific to adult migraine patients treated with atogepant for 12 weeks comparing with placebo and only for double-blind phase of RCTs.

**(D) Imprecision**: Focusing on width of the confidence interval; when the study includes a small sample size and a low incidence rate of outcomes, resulting in a wider credible interval, the quality rating of evidence will be downgraded.

**(E) Publication Bias:** We conducted a comprehensive search on the clinical trials registration website (clinicaltrials.gov) to identify and consider any potentially unpublished evidence. Meanwhile, we carefully review the research methods and results, as well as the funding sources of the study, the research backgrounds of the authors, and whether there are any conflicts of interest, to determine whether there are factors that may lead to publication bias.

Supplementary Table 1 Summary of GRADE results for each outcomes

| Medications  Outcomes | Atogepant | Atogepant 10mg QD | Atogepant 30mg QD | Atogepant 60mg QD | Atogepant 30mg BID | Atogepant 60mg BID |
| --- | --- | --- | --- | --- | --- | --- |
| MMDs | high | high | high | moderat^1^ | high | low^2, 3^ |
| MMDs (week 1-4) | high | high | high | moderat^1^ | high | low^2, 3^ |
| MMDs (week 5-8) | high | high | high | high | high | low^2, 3^ |
| MMDs (week 9-12) | high | high | moderate^1^ | high | high | low^2, 3^ |
| MHDs | high | high | high | moderate^1^ | high | low^2, 3^ |
| Acute medication use days | high | high | high | high | high | low^2, 3^ |
| 50% responder rate | high | high | high | high | high | low^2, 3^ |
| TEAEs | moderate^1^ | moderate^1^ | moderate^1^ | moderate^1^ | high | low^2, 3^ |
| Treatment-related TEAEs | moderate^1^ | moderate^2^ | moderate^1^ | moderate^1^ | moderate^2^ | low^2, 3^ |
| Serious TEAEs | moderate^2^ | moderate^2^ | low^1, 2^ | moderate^2^ | moderate^2^ | very low^1, 2, 3^ |
| Constipation | high | moderate^2^ | high | high | high | low^2, 3^ |
| Nausea | high | moderate^2^ | moderate^2^ | high | high | low^2, 3^ |
| Fatigue | high | low^1, 2^ | low^1, 2^ | moderate^2^ | moderate^2^ | very low^1, 2, 3^ |
| Urinary tract infection | high | low^1, 2^ | moderate^2^ | moderate^2^ | moderate^2^ | low^2, 3^ |
| Nasopharyngitis | high | low^1, 2^ | moderate^2^ | moderate^2^ | llow^1, 2^ | low^2, 3^ |
| Upper respiratory tract infection | moderate^1^ | low^1, 2^ | low^1, 2^ | low^1, 2^ | low^1, 2^ | very low^1, 2, 3^ |

1 downgraded due to inconsistency

2 downgraded due to imprecision

3 downgraded due to publication bias

Ato Atogepant

QD quaque die, once daily

BID both in die, twice daily

MMDs Monthly migraine days

MHDs Monthly headache days

TEAEs treatment-emergent adverse events
