# Supplementary material for: Efficacy and safety of atogepant, a small molecule CGRP receptor antagonist, for the preventive treatment of migraine: a systematic review and meta-analysis
Source: J Headache Pain. 2024 Jul 19;25(1):116. doi: 10.1186/s10194-024-01822-2 (PMC11264921; doi:10.1186/s10194-024-01822-2)
Supplement: Supplementary file 3 — Supplementary Material 3 [file 10194_2024_1822_MOESM3_ESM.doc]

**Supplementary Figures**


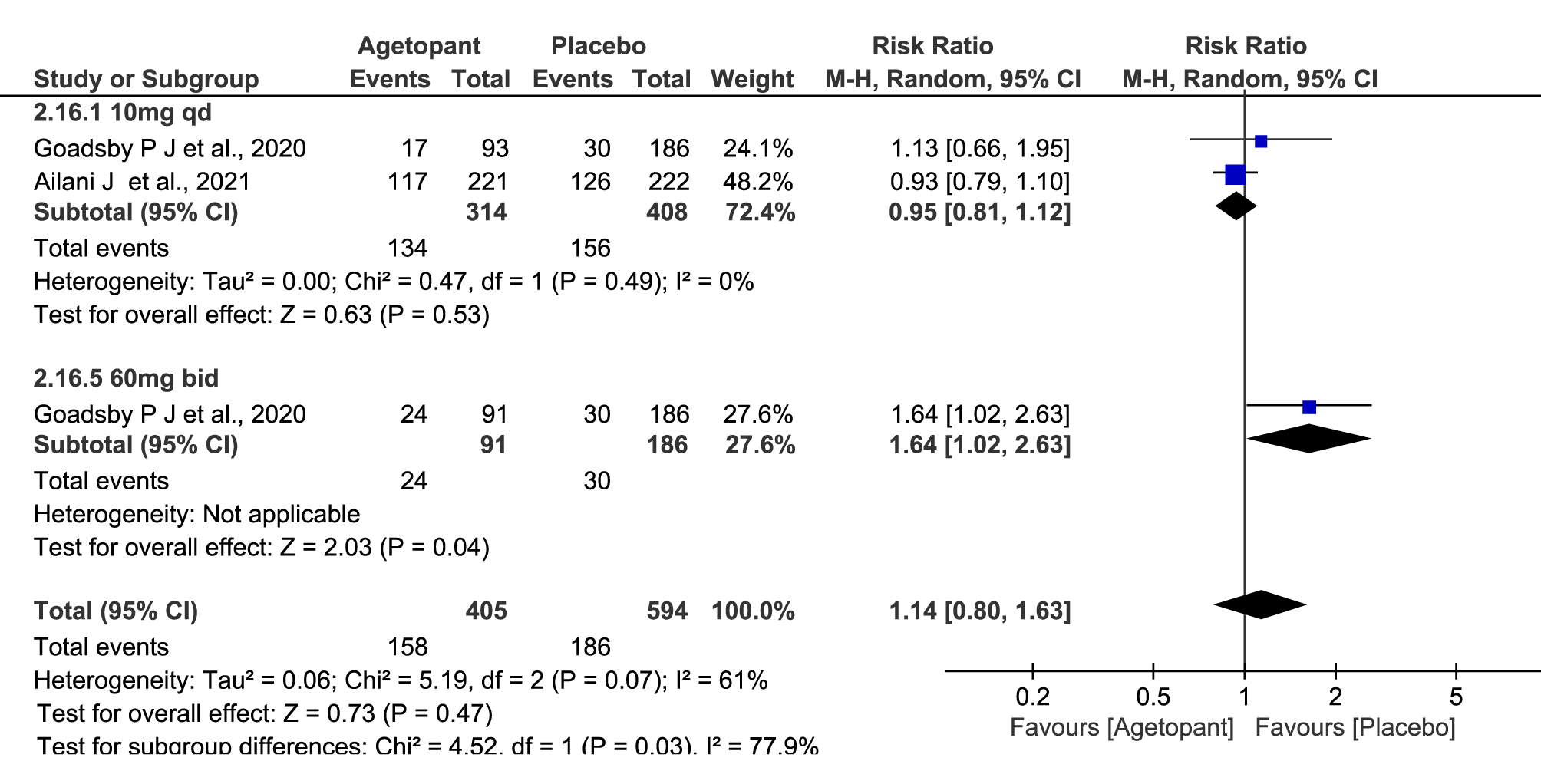


**Supplementary Figure 1** Meta-analysis of the treatment related TEAEs after therapy with atogepant 10 mg QD or 60 mg BID compared with placebo. The diamond indicates the estimated relative risk with 95% confidence interval for the pooled patients. M-H, Mantel-Haenszel; CI, confidence interval.


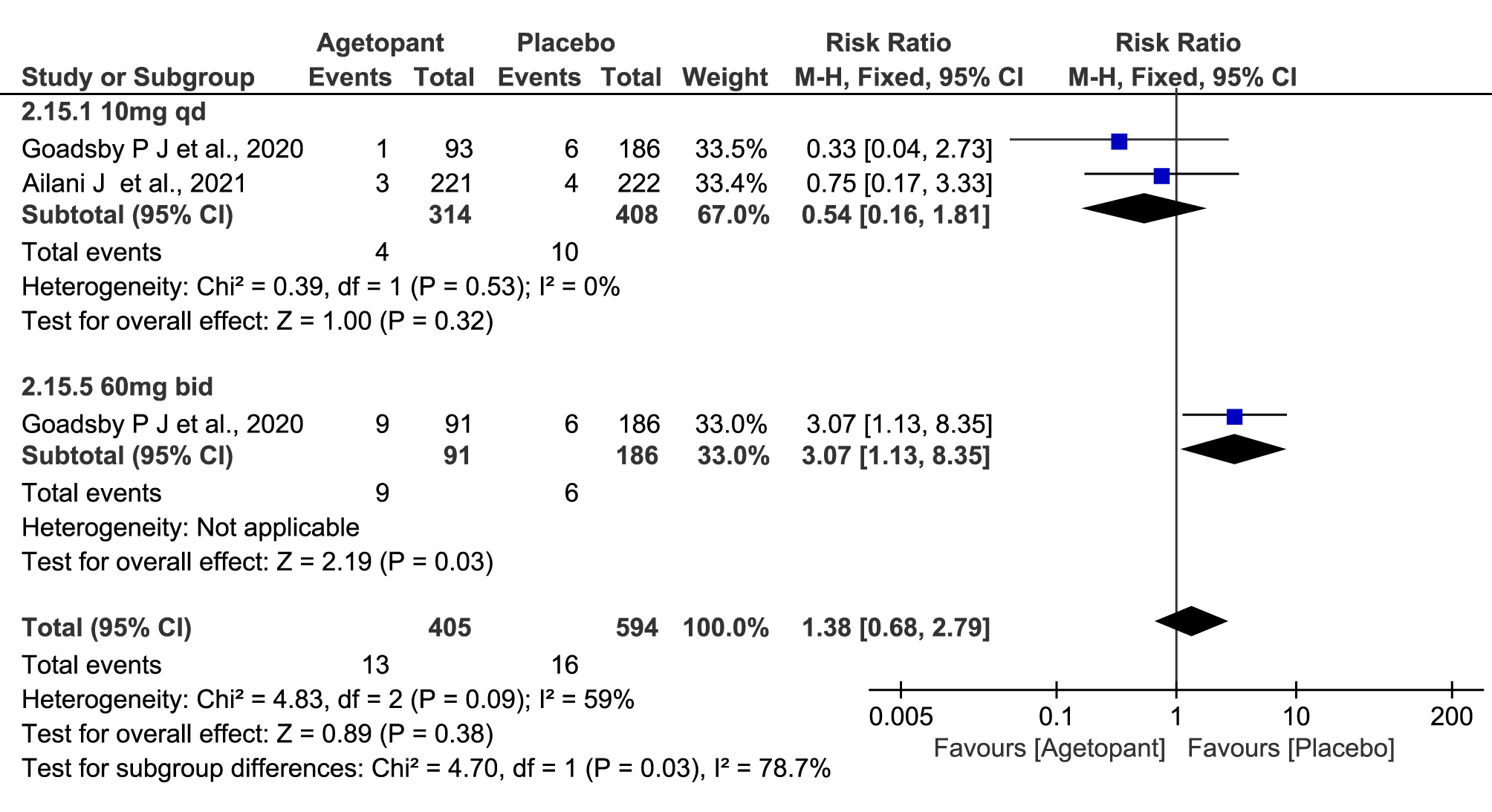


**Supplementary Figure 2** Meta-analysis of fatigue after therapy with atogepant 10 mg QD or 60 mg BID compared with placebo. The diamond indicates the estimated relative risk with 95% confidence interval for the pooled patients. M-H, Mantel-Haenszel; CI, confidence interval.


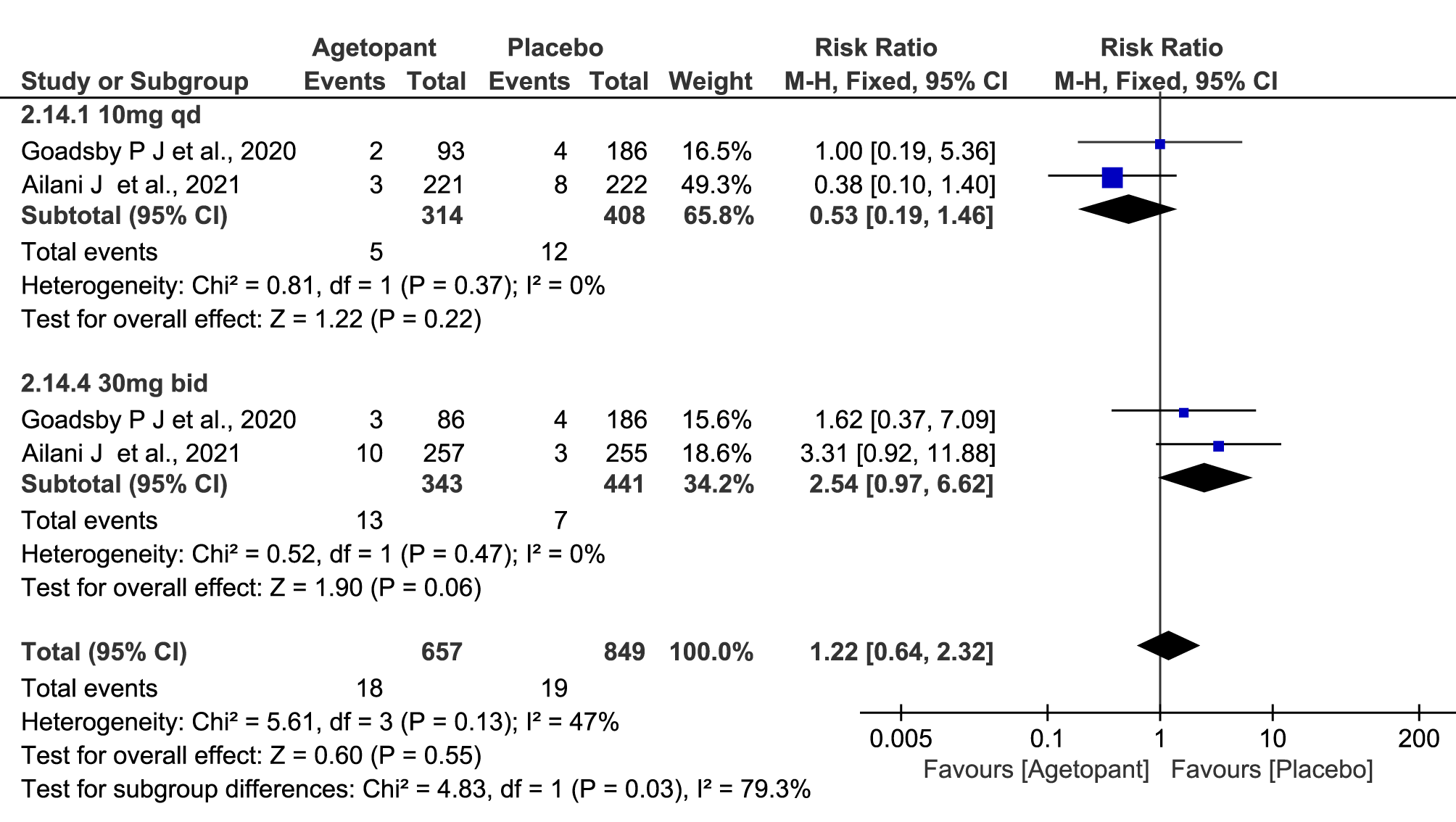


**Supplementary Figure 3** Meta-analysis of urinary tract infection after therapy with atogepant 10 mg QD or 30 mg BID compared with placebo. The diamond indicates the estimated relative risk with 95% confidence interval for the pooled patients. M-H, Mantel-Haenszel; CI, confidence interval.


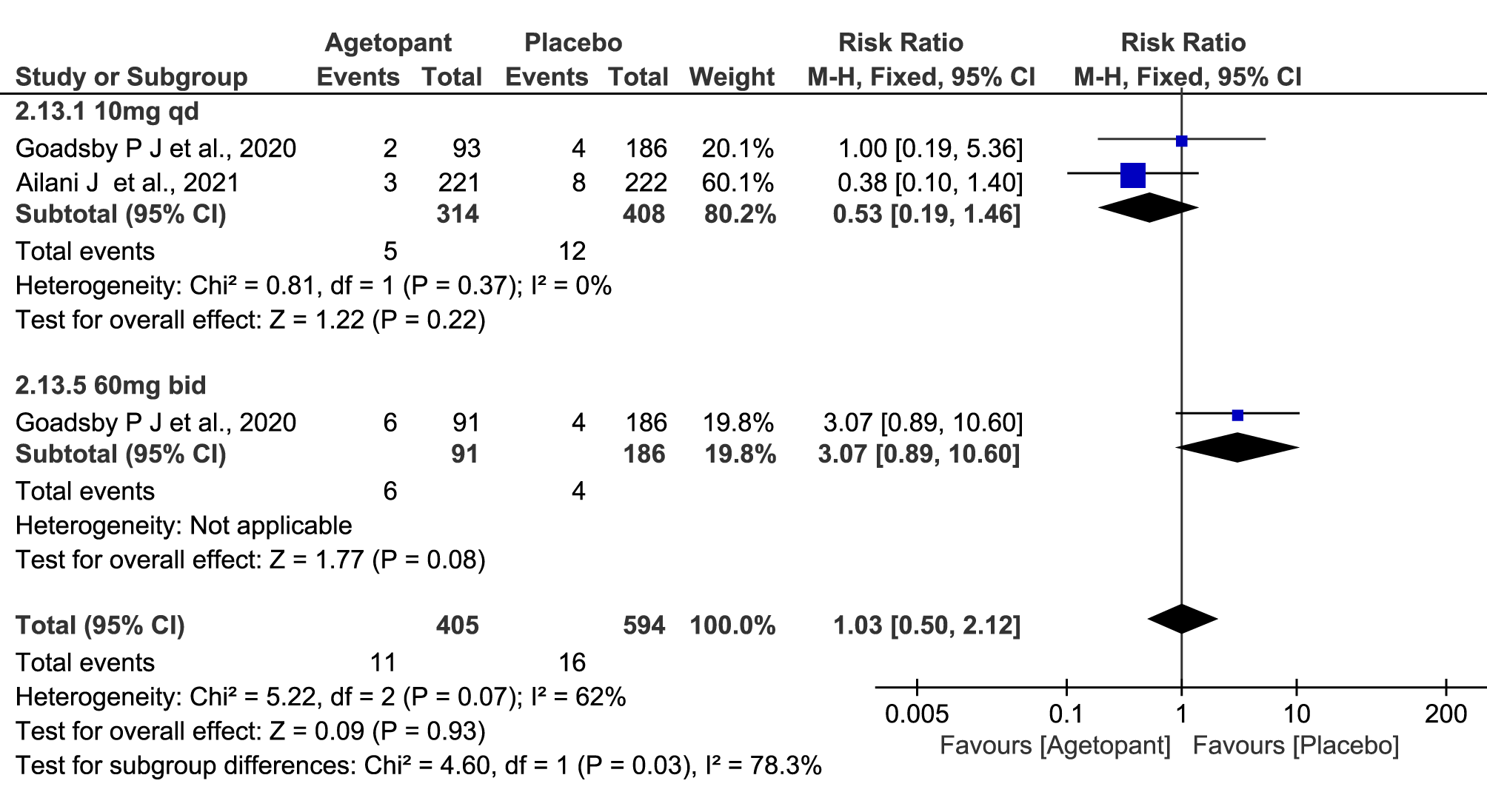


**Supplementary Figure 4** Meta-analysis of urinary tract infection after therapy with atogepant 10 mg QD or 60 mg BID compared with placebo. The diamond indicates the estimated relative risk with 95% confidence interval for the pooled patients. M-H, Mantel-Haenszel; CI, confidence interval.
